# Supplementary material for: Structure–Activity Relationship Studies of 9-Alkylamino-1,2,3,4-tetrahydroacridines against Leishmania (Leishmania) infantum Promastigotes
Source: Pharmaceutics. 2023 Feb 16;15(2):669. doi: 10.3390/pharmaceutics15020669 (PMC9965875; doi:10.3390/pharmaceutics15020669)
Supplement: Supplementary file 1 [file pharmaceutics-15-00669-s001.zip › pharmaceutics-2142347-supplementary.pdf]

Supplementary information:

Synthesis of 9-chloroacridines (5):

9-chloro-1,2,3,4-tetrahydroacridine ***5.a***. Brown solid, m.p. 67-69 ˚C (68-70 ˚C)[15], yield 41 % [*Conv.*] or 61 % [*MW*]. ^{1}H NMR (300 MHz, $CDCl_3$): δ 1.87-1.99 (4H, m, H-2,3), 2.98 (2H, t, J = 6.2 Hz, H-1), 3.11 (2H, t, J = 6.2 Hz, H-4), 7.51 (1H, ddd, J = 8.2, 6.9 and 1.3 Hz, H-7), 7.64 (1H, ddd, J = 8.4, 6.8 and 1.5 Hz, H-6), 7.96 (1H, d, J = 8.0 Hz, H-5), 8.13 (1H, dd, J = 8.4 and 1.4 Hz, H-8). ^{13}C NMR (75 MHz, $CDCl_3$): δ 22.6-22.7 (C-2,3), 27.5 (C-1), 34.2 (C-4), 123.6 (C-8), 125.3 (C-8a), 126.4 (C-7), 128.6 (C-6), 128.8 (C-9a), 129.2 (C-5), 141.4 (C-9), 146.6 (C-10a), 159.4 (C-4a). HRMS-ESI [m/z]: Calculated for $C_{13}H_{13}ClN$ $[M+H]^+$: 218.0731; Determined 218.0730.

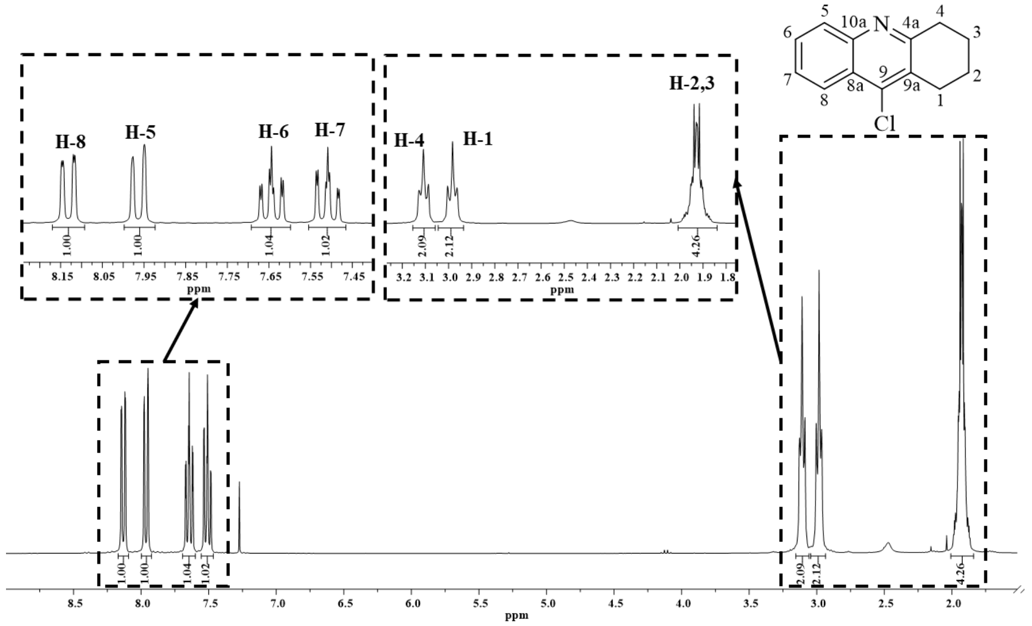

Figure S1. *^{1}H NMR spectrum of 9-chloro-1,2,3,4-tetrahydroacridine (**5.a**).*

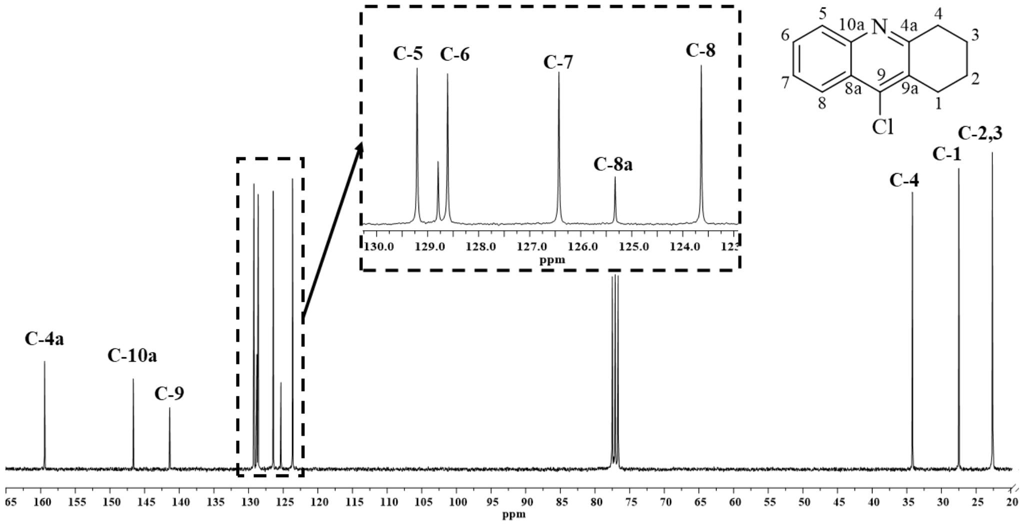

Figure S2. *^{13}C NMR spectrum of 9-chloro-1,2,3,4-tetrahydroacridine (**5.a**).*

6,9-dichloro-1,2,3,4-tetrahydroacridine **5.b**. Light brown solid, m.p. 81-84 ˚C (81-83 ˚C) [15], yield 68 % [Conv.] or 71 % [MW]. ^{1}H NMR (300 MHz, $CDCl_3$): δ 1.85-2.02 (4H, m, H-2,3), 2.98 (2H, t, J = 6.5 Hz, H-1), 3.09 (2H, t, J = 6.5 Hz, H-4), 7.45 (1H, dd, J = 9.0 and 2.1 Hz, H-7), 7.95 (1H, d, J = 2.0 Hz, H-5), 8.05 (1H, d, J = 8.9 Hz, H-8). ^{13}C NMR (75 MHz, $CDCl_3$): δ 22.5 (C-2,3), 27.5 (C-1), 34.2 (C-4), 123.8 (C-8a), 125.1 (C-8), 127.4 (C-7), 127.6 (C-5), 129.2 (C-9a), 135.1 (C-6), 141.4 (C-9), 146.9 (C-10a), 160.8 (C-4a). HRMS-ESI [m/z]: Calculated for $C_{13}H_{12}Cl_2N$ $[M+H]^+$: 252.0341; Determined 252.0340.

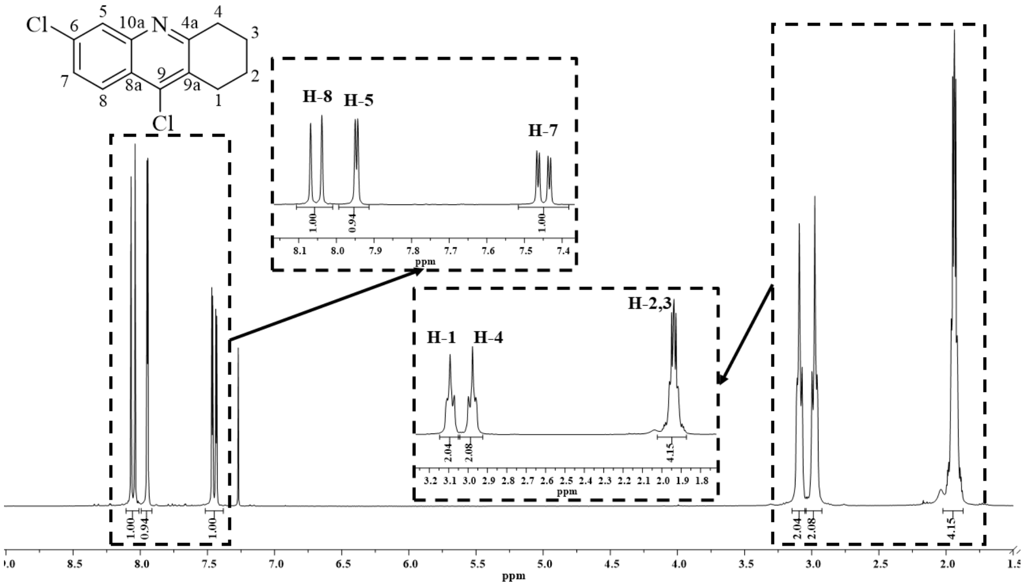

Figure S3. *^{1}H NMR spectrum of 6,9-dichloro-1,2,3,4-tetrahydroacridine (**5.b**)*

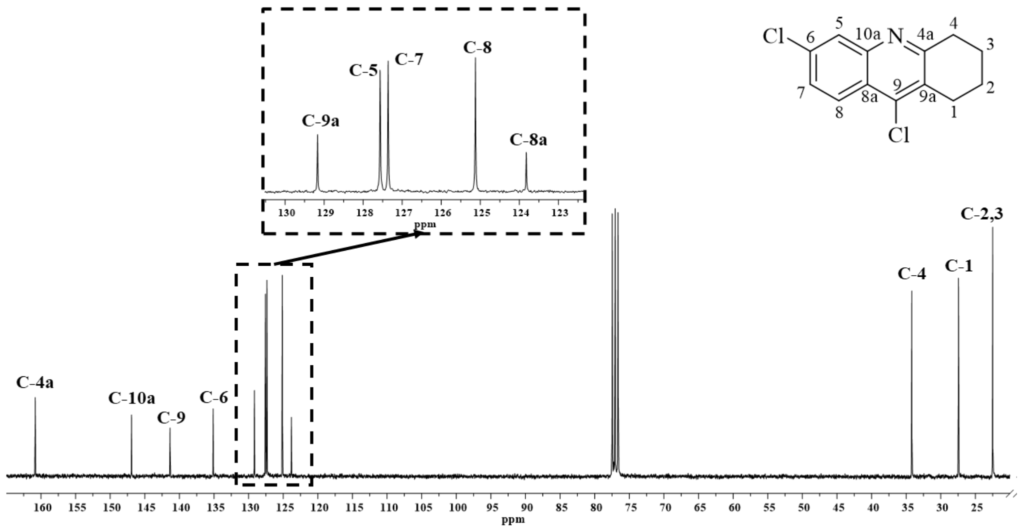

Figure S4. *^{13}C NMR spectrum of 6,9-dichloro-1,2,3,4-tetrahydroacridine (**5.b**)*

Synthesis of 9-alkylamino-1,2,3,4-tetrahydroacridines (6.a-n):

Derivative **6.h as example of NMR spectra.*

N[1]*-(1,2,3,4-tetrahydroacridin-9-yl)ethane-1,2-diamine* ***6.a***, Brown solid, m.p. 125-136 ˚C, yield 61 %. ^{1}H NMR (300 MHz, CD_3OD): *δ* 1.87-1.95 (4H, m, H-2,3), 2.74-2.81 (2H, m, H-1), 2.93 (2H, t, *J* = 6.5 Hz, H-2'), 2.96-3.02 (2H, m, H-4), 3.60 (2H, t, *J* = 6.5 Hz, H-1'), 7.40 (1H, ddd, *J* = 8.4, 6.8 and 1.3 Hz, H-7), 7.58 (1H, ddd, *J* = 8.4, 6.8 and 1.4 Hz, H-6), 7.78 (1H, dd, *J* = 8.5 and 1.3 Hz, H-5), 8.13 (1H, dd, *J* = 8.4 and 1.4 Hz, H-8). ^{13}C NMR (75 MHz, CD_3OD): δ 22.1 (C-2), 22.6 (C-3), 24.6 (C-1), 32.4 (C-4), 41.6 (C-2'), 50.0 (C-1'), 115.9 (C-9a), 119.8 (C-8a), 123.0 (C-8), 123.7 (C-7), 125.9 (C-5), 128.8 (C-6), 145.8 (C-10a), 152.1 (C-9), 157.3 (C-4a). HRMS-ESI [m/z]: Calculated for $C_{15}H_{20}N_3$ $[M+H]^+$: 242.1652; Determined 242.1647.

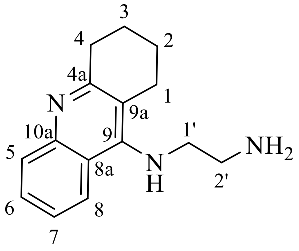

Figure S5. *N*[1]*-(1,2,3,4-tetrahydroacridin-9-yl)ethane-1,2-diamine (**6.a**).*

N[1]*-(1,2,3,4-tetrahydroacridin-9-yl)propane-1,3-diamine* ***6.b***. Brown oil, yield 58 %. ^{1}H NMR (300 MHz, CD_3OD): *δ* 1.78 (2H, p, *J* = 7.1 Hz, H-2'), 1.81-1.92 (4H, m, H-2,3), 2.70 (4H, t, *J* = 7.0 Hz, H-3',1), 2.95 (2H, t, *J* = 5.9 Hz, H-4), 3.56 (2H, t, *J* = 7.1 Hz, H-1'), 7.35 (1H, ddd, *J* = 8.4, 6.8 and 1.3 Hz, H-7), 7.54 (1H, ddd, *J* = 8.4, 6.8 and 1.4 Hz, H-6), 7.77 (1H, dd, *J* = 8.6 and 1.3 Hz, H-5), 8.09 (1H, dd, *J* = 8.4 and 1.3 Hz, H-8). ^{13}C NMR (75 MHz, CD_3OD): *δ* 23.6 (C-2), 24.0 (C-3), 26.2 (C-1), 34.1 (C-4), 34.6 (C-2'), 40.1 (C-3'), 47.3 (C-1'), 116.9 (C-9a), 121.2 (C-8a), 124.3 (C-8), 124.8 (C-7), 127.8 (C-5), 129.8 (C-6), 147.7 (C-10a), 153.0 (C-9), 159.0 (C-4a). HRMS-ESI [m/z]: Calculated for $C_{16}H_{22}N_3$ $[M+H]^+$: 256.1808; Determined 256.1816.

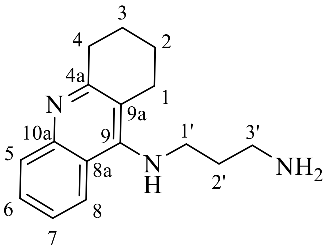

Figure S6. *N*[1]*-(1,2,3,4-tetrahydroacridin-9-yl)propane-1,3-diamine (**6.b**).*

N[1]*-(1,2,3,4-tetrahydroacridin-9-yl)butane-1,4-diamine* ***6.c***. Brown oil, yield 43 %. ^{1}H NMR (500 MHz, CD_3OD): δ 1.61-1.69 (2H, m, H-2′), 1.69-1.77 (2H, m, H-3′), 1.88-1.96 (4H, m, H-2,3), 2.75-2.79 (2H, m, H-1), 2.81 (2H, t, *J* = 7.3 Hz, H-4′), 3.00 (2H, t, *J* = 5.9 Hz, H-4), 3.62 (2H, t, *J* = 7.0 Hz, H-1′), 7.41 (1H, ddd, *J* = 8.3, 6.8 and 1.3 Hz, H-7), 7.60 (1H, ddd, *J* = 8.3, 6.8 and 1.3 Hz, H-6), 7.79 (1H, dd, *J* = 8.5 and 1.3 Hz, H-5), 8.15 (1H, dd, *J* = 8.6 and 1.3 Hz, H-8). ^{13}C NMR (125 MHz, CD_3OD): δ 22.1 (C-2), 22.6 (C-3), 24.7 (C-1), 27.0 (C-2′), 27.9 (C-3′), 32.2 (C-4), 39.9 (C-4′), 48.12 (C-1′), 115.2 (C-9a), 119.5 (C-8a), 123.2 (C-8), 123.6 (C-7), 125.7 (C-5), 128.9 (C-6), 145.6 (C-10a), 152.3 (C-9), 157.0 (C-4a). HRMS-ESI [m/z]: Calculated for $C_{17}H_{24}N_3$ $[M+H]^+$: 270.1965; Determined 270.1973.

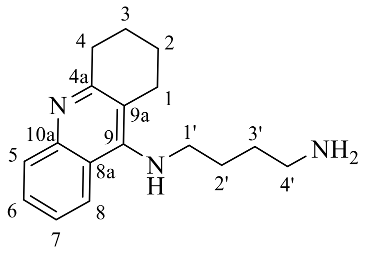

Figure S7. *N*[1]*-(1,2,3,4-tetrahydroacridin-9-yl)butane-1,4-diamine (**6.c**).*

N[1]*-(1,2,3,4-tetrahydroacridin-9-yl)pentane-1,5-diamine* ***6.d***. Brown oil, yield 53 %. ^{1}H NMR (300 MHz, CD_3OD): δ 1.37-1.50 (2H, m, H-3′), 1.52-1.64 (2H, m, H-4′), 1.71 (2H, p, *J* = 7.4 Hz; H-2′), 1.85-2.00 (4H, m, H-2,3), 2.77 (2H, t, *J* = 7.4 Hz, H-5′), 2.77 (2H, broad s, H-1), 3.00 (2H, broad s, H-4), 3.60 (2H, t, *J* = 7.4 Hz, H-1′), 7.40 (1H, ddd, *J* = 8.4, 6.8 and 1.3 Hz, H-7), 7.60 (1H, ddd, *J* = 8.4, 6.8 and 1.4 Hz, H-6), 7.78 (1H, dd, *J* = 8.4 and 1.2 Hz; H-5), 8.14 (1H, dd, *J* = 8.4 and 1.3 Hz, H-8). ^{13}C NMR (75 MHz, CD_3OD): δ 22.1 (C-3′), 22.6 (C-2), 23.6 (C-3), 24.7 (C-4), 29.3 (C-4′), 30.4 (C-2′), 32.3 (C-1), 40.0 (C-5′), 48.1 (C-1′), 115.1 (C-9a), 119.5 (C-8a), 123.2 (C-8), 123.5 (C-7), 125.7 (C-5), 128.8 (C-6), 145.6 (C-10a), 152.3 (C-9), 156.9 (C-4a). HRMS-ESI [m/z]: Calculated for $C_{18}H_{26}N_3$ $[M+H]^+$: 284.2121; Determined: 284.2139.

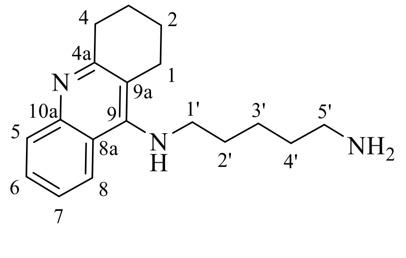

Figure S8. *N*[1]*-(1,2,3,4-tetrahydroacridin-9-yl)pentane-1,5-diamine (**6.d**).*

N[1]*-(1,2,3,4-tetrahydroacridin-9-yl)octane-1,8-diamine* ***6.e***. Brown oil, yield 69 %. ^{1}H NMR (500 MHz, $CDCl_3$): δ 1.08-1.25 (8H, m, H-3′,4′,5′,6′), 1.35-1.46 (2H, m, H-7′), 1.50 (2H, p, *J* = 7.3 Hz, H-2′), 1.71-1.85 (4H, m, H-2,3), 2.55 (2H, d, *J* = 5.4 Hz, H-1), 2.64 (2H, t, *J* = 7.4 Hz, H-8′), 2.94 (2H, t, *J* = 6.1 Hz,

H-4), 3.34 (2H, t, *J* = 7.3 Hz, H-1′), 2.94 (2H, t, *J* = 6.1 Hz, H-4), 3.34 (2H, t, *J* = 7.3 Hz, H-1′), 7.22 (1H, ddd, *J* = 8.4, 6.7 and 1.3 Hz, H-7), 7.42 (1H, ddd, *J* = 8.4, 6.7 and 1.3 Hz, H-6), 7.80 (1H, dd, *J* = 8.4 and 1.3 Hz, H-5), 7.85 (1H, dd, *J* = 8.4 and 1.3 Hz, H-8). ^{13}C NMR (125 MHz, $CDCl_3$): δ 22.7 (C-2), 23.0 (C-3), 24.7 (C-1), 26.6 (C-6′), 26.7 (C-3′), 29.2 (C-4′,5′), 31.2 (C-7′), 31.6 (C-2′), 33.7 (C-4), 40.9 (C-8′), 49.2 (C-1′), 115.5 (C-9a), 120.0 (C-8a), 122.9 (C-8), 123.5 (C-7), 128.2 (C-5), 128.3 (C-6), 147.1 (C-10a), 150.9 (C-9), 158.1 (C-4a). HRMS-ESI [m/z]: Calculated for $C_{21}H_{32}N_3$ $[M+H]^+$: 326.2591; Determined: 326.2608.

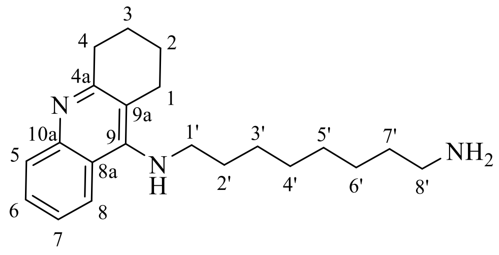

Figure S9. *N^1-(1,2,3,4-tetrahydroacridin-9-yl)octane-1,8-diamine (**6.e**).*

N^1-(1,2,3,4-tetrahydroacridin-9-yl)decane-1,10-diamine ***6.f***. Brown oil, yield 65 %. ^1H NMR (300 MHz, CD_3OD): δ 1.19-1.39 (8H, m, H-4′,5′,6′,7′), 1.44-1.56 (2H, m, H-8′), 1.63 (2H, p, *J* = 7.1 Hz, H-3′), 1.87-1.95 (4H, m, H-2,3), 2.64-2.73 (2H, m, H-10′), 2.71-2.80 (2H, m, H-4), 2.95-3.02 (2H, m, H-1), 3.53 (2H, t, *J* = 7.1 Hz, H-1′), 7.37 (1H, ddd, *J* = 8.3, 6.8 and 1.3 Hz, H-7), 7.56 (1H, ddd, *J* = 8.4, 6.8 and 1.4 Hz, H-6), 7.78 (1H, dd, *J* = 8.8 and 1.1 Hz, H-5), 8.10 (1H, dd, *J* = 8.5 and 1.1 Hz, H-8). ^{13}C NMR (75 MHz, CD_3OD): δ 22.3 (C-3), 22.7 (C-2), 24.8 (C-4), 26.4 (C-9′), 26.5 (C-2′), 28.9-29.1 (C-4′,5′,6′,7′), 30.7 (C-8′), 30.9 (C-3′), 32.7 (C-1), 40.6 (C-10′), 48.3 (C-1′), 115.2 (C-9a), 119.8 (C-8a), 123.1 (C-8), 123.3 (C-7), 126.4 (C-5), 128.4 (C-6), 146.4 (C-10a), 152.0 (C-9), 157.5 (C-4a). HRMS-ESI [m/z]: Calculated for $C_{23}H_{36}N_3$ $[M+H]^+$: 354.2904; Determined: 354.2912.

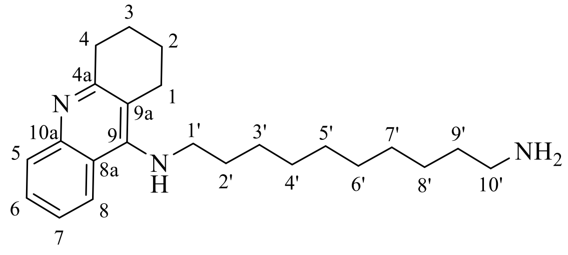

Figure S10. *N^1-(1,2,3,4-tetrahydroacridin-9-yl)decane-1,10-diamine (**6.f**).*

N^1-(1,2,3,4-tetrahydroacridin-9-yl)dodecane-1,12-diamine ***6.g***. Brown oil, yield 73 %. ^1H NMR (300 MHz, CD_3OD): δ 1.14-1.39 (16H, m, H-2',4',5',6',7',8',9',11'), 1.54 (2H, p, *J* = 7.5 Hz, H-10′), 1.62 (2H, p, *J* = 7.5 Hz, H-3′), 1.81-1.93 (4H, m, H-2,3), 2.72 (2H, broad s, H-1), 2.75 (2H, t, *J* = 7.2 Hz, H-12′), 2.98 (2H, broad s, H-4), 3.53 (2H, t, *J* = 7.2 Hz, H-1′), 7.36 (1H, ddd, *J* = 8.4, 6.8 and 1.3 Hz, H-7),

7.56 (1H, ddd, *J* = 8.4, 6.8 and 1.3 Hz, H-6), 7.78 (1H, dd, *J* = 8.4 and 1.2 Hz, H-5), 8.09 (1H, dd, *J* = 8.4 and 1.3 Hz, H-8). ^{13}C NMR (75 MHz, CD_3OD): δ 22.2 (C-2), 22.7 (C-3), 24.7 (C-4), 26.4 (C-11′), 26.5 (C-2′), 29.0 (C-8′), 29.1 (C-5′), 29.2-29.3 (C4′,6′,7′,9′), 30.0 (C-10′), 30.9 (C-3′), 32.5 (C-1), 40.3 (C-12′), 48.3 (C-1′), 115.0 (C-9a), 119.7 (C-8a), 123.1 (C-8), 123.3 (C-7), 126.2 (C-5), 128.5 (C-6), 146.1 (C-10a), 152.1 (C-9), 157.2 (C-4a). HRMS-ESI [m/z]: Calculated for $C_{25}H_{40}N_3$ $[M+H]^+$: 382.3217; Determined: 382.3239.

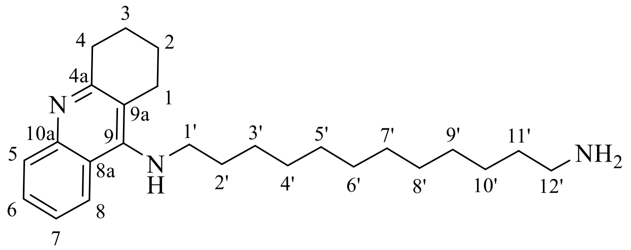

Figure S11. *N^1-(1,2,3,4-tetrahydroacridin-9-yl)dodecane-1,12-diamine (**6.g**).*

N^1-(6-chloro-1,2,3,4-tetrahydroacridin-9-yl)ethane-1,2-diamine ***6.h***, m.p. 296-300 ˚C, yield 46 %. ^{1}H NMR (300 MHz, CD_3OD): δ 1.91 (4H, p, *J* = 3.3 Hz, H-2,3), 2.72-2.79 (2H, m, H-4), 2.92 (2H, t, *J* = 6.5 Hz, H-2′), 2.95-3.00 (2H, m, H-1), 3.58 (2H, t, *J* = 6.5 Hz, H-1′), 7.33 (1H, dd, *J* = 9.1 and 2.2 Hz, H-7), 7.74 (1H, d, *J* = 2.2 Hz, H-5), 8.09 (1H, d, *J* = 9.1 Hz, H-8). ^{13}C NMR (75 MHz, CD3OD): δ 22.1 (C-3), 22.5 (C-2), 24.6 (C-4), 32.9 (C-1), 41.7 (C-2′), 50.2 (C-1′), 116.3 (C-9a), 118.3 (C-8a), 123.9 (C-7), 125.0 (C-8), 125.3 (C-5), 134.1 (C-6), 147.1 (C-10a), 151.8 (C-9), 159.2 (C-4a). HRMS-ESI [m/z]: Calculated for $C_{15}H_{18}ClN_3$ $[M+H]^+$: 276.1262; Determined: 276.1281.

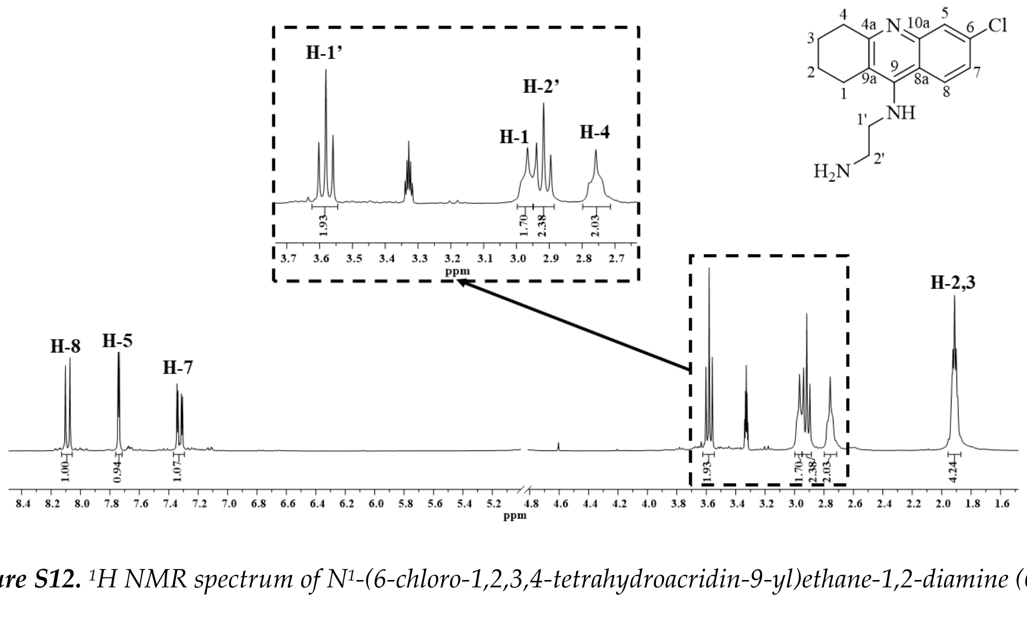

Figure S12. *^{1}H NMR spectrum of N^1-(6-chloro-1,2,3,4-tetrahydroacridin-9-yl)ethane-1,2-diamine (**6.h**).*

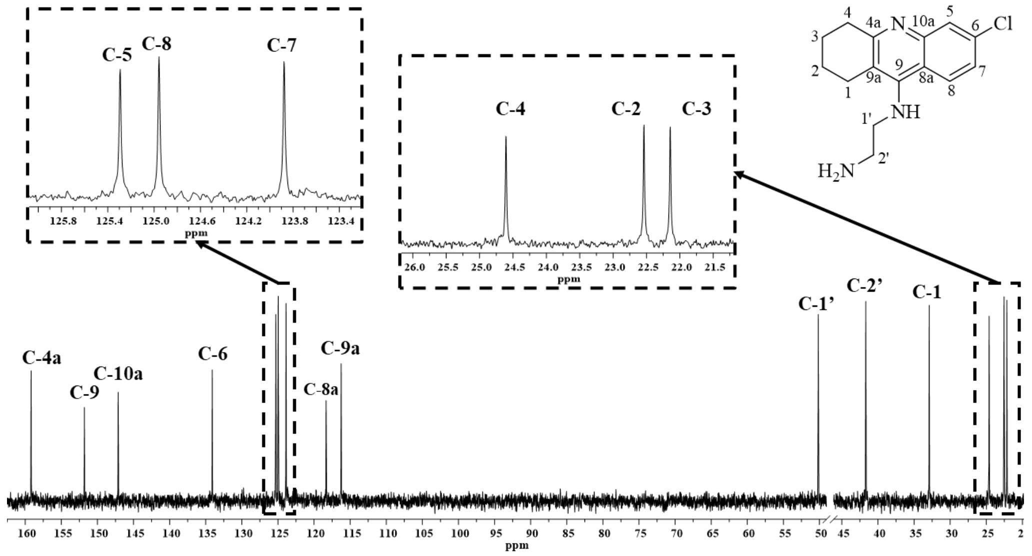

Figure S13. *^{13}C NMR spectrum of N[1]-(6-chloro-1,2,3,4-tetrahydroacridin-9-yl)ethane-1,2-diamine (**6.h**).*

N^1-(6-chloro-1,2,3,4-tetrahydroacridin-9-yl)propane-1,3-diamine ***6.i***. Yellow solid, m.p. 179-182 ˚C, yield 48 %. ^{1}H NMR (300 MHz, CD_3OD): δ 1.92-1.99 (4H, m, H-2,3), 2.09 (2H, p, J = 7.6 Hz, H-2′), 2.76 (2H, broad s, H-1), 3.03 (2H, broad s, H-4), 3.04 (2H, t, J = 7.1 Hz, H-3′), 3.85 (2H, t, J = 7.1 Hz, H-1′), 7.49 (1H, dd, J = 9.2 and 2.2 Hz, H-7), 7.80 (1H, d, J = 2.2 Hz, H-5), 8.26 (1H, d, J = 7.2 Hz, H-8). 13C NMR (75 MHz, CD_3OD): δ 21.3 (C-3), 22.0 (C-2), 24.3 (C-1), 28.4 (C-4), 30.6 (C-2′), 36.9 (C-3′), 44.8 (C-1′), 114.7 (C-9a), 116.5 (C-8a), 121.9 (C-5), 124.9 (C-7), 125.8 (C-8), 136.3 (C-6), 143.3 (C-10a), 153.7 (C-9), 155.6 (C-4a). HRMS-ESI [m/z]: Calculated for $C_{16}H_{21}ClN_3$ $[M+H]^+$: 290.1419; Determined: 290.1424.

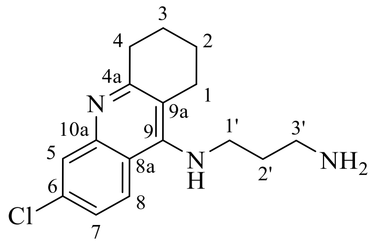

Figure S14. *N^1-(1,2,3,4-tetrahydroacridin-9-yl)propane-1,3-diamine (**6.i**).*

N^1-(6-chloro-1,2,3,4-tetrahydroacridin-9-yl)butane-1,4-diamine ***6.j***. Brown solid, 115-120 ˚C, yield 55 %. ^{1}H NMR (300 MHz, CD_3OD): δ 1.63-1.77 (4H, m, H-2′,3′), 1.87-1.93 (4H, m, H-2,3), 2.68-2.76 (2H, m, H-1), 2.87 (2H, t, J = 7.2 Hz, H-4′), 2.91-2.98 (2H, m, H-4), 3.59 (2H, t, J = 6.7 Hz, H-1′), 7.30 (1H, dd, J = 9.1 and 2.2 Hz, H-7), 7.72 (1H, d, J = 2.2 Hz, H-5), 8.07 (1H, d, J = 9.1 Hz, H-8). ^{13}C NMR (75 MHz, CD_3OD): δ 21.9 (C-2), 22.4 (C-3), 24.5 (C-1), 25.7 (C-3′), 27.7 (C-2′), 32.6 (C-4), 39.3 (C-4′), 47.5 (C-1′), 115.4 (C-9a), 117.8 (C-8a), 123.6 (C-7), 124.9 (C-5), 124.9 (C-8), 134.0 (C-6), 146.7 (C-10a), 151.6 (C-9), 158.6 (C-4a). HRMS-ESI [m/z]: Calculated for $C_{17}H_{23}ClN_3$ $[M+H]^+$: 304.1575; Determined: 304.1591.

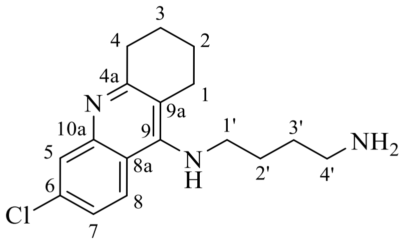

Figure S15. *N^1-(6-chloro-1,2,3,4-tetrahydroacridin-9-yl)butane-1,4-diamine (**6.j**).*

N^1-(6-chloro-1,2,3,4-tetrahydroacridin-9-yl)pentane-1,5-diamine ***6.k***. Brown solid, m.p. 68-74 ˚C, yield 31 %. ^{1}H NMR (300 MHz, CD_3OD): δ 1.38-1.51 (2H, m, H-3′), 1.54-1.65 (2H, m, H-4′), 1.62-1.78 (2H, m, H-2′), 1.88-1.95 (4H, m, H-2,3), 2.74 (2H, broad s, H-1), 2.80 (2H, t, J = 7.5 Hz, H-5′), 2.97 (2H, broad s, H-4), 3.58 (2H, t, J = 7.2 Hz, H-1′), 7.33 (1H, dd, J = 9.2 and 2.2 Hz, H-7), 7.74 (1H, d, J = 2.1 Hz, H-5), 8.10 (1H, d, J = 9.2 Hz, H-8). ^{13}C NMR (75 MHz, CD_3OD): δ 22.2 (C-2), 22.6 (C-3), 23.6

(C-3′), 24.7 (C-1), 28.7 (C-4′), 30.4 (C-2′), 32.8 (C-4), 39.8 (C-5′), 48.1 (C-1′), 115.5 (C-9a), 118.1 (C-8a), 123.7 (C-7), 125.1 (C-8), 125.2 (C-5), 134.1 (C-6), 147.1 (C-10a), 151.9 (C-9), 159.0 (C-4a). HRMS-ESI [m/z]: Calculated for $C_{18}H_{25}ClN_3$ $[M+H]^+$: 318.1732; Determined 318.1727.

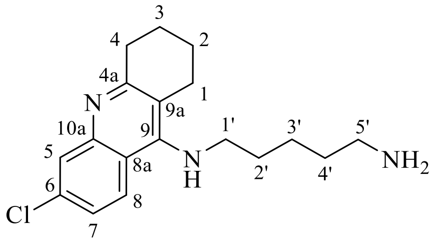

Figure S16. *N¹-(6-chloro-1,2,3,4-tetrahydroacridin-9-yl)pentane-1,5-diamine (**6.k**).*

N^1-(6-chloro-1,2,3,4-tetrahydroacridin-9-yl)octane-1,8-diamine ***6.l***. Brown oil, yield 50 %. ^{1}H NMR (300 MHz, CD_3OD): δ 1.22-1.43 (8H, m, H-2′,4′,5′,7′), 1.54-1.72 (4H, m, H-3′,6′), 1.83-1.97 (4H, m, H-2,3), 2.70 (2H, broad s, H-1), 2.87 (2H, t, *J* = 7.4 Hz, H-8′), 2.93-3.00 (2H, m, H-4), 3.57 (2H, t, *J* = 7.2 Hz, H-1′), 7.32 (1H, dd, *J* = 9.1 and 2.2 Hz, H-7), 7.74 (1H, d, *J* = 2.2 Hz, H-5), 8.09 (1H, d, *J* = 9.2 Hz, H-8). ^{13}C NMR (75 MHz, CD_3OD): δ 22.0 (C-2), 22.5 (C-3), 24.5 (C-1), 26.0 (C-7′), 26.4 (C-2′), 27.6 (C-6′), 28.7 (C-4′), 28.8 (C-5′), 30.7 (C-3′), 32.5 (C-4), 39.5 (C-8′), 48.2 (C-1′), 115.0 (C-9a), 117.7 (C-8a), 123.7 (C-7), 124.6 (C-5), 125.3 (C-8), 134.4 (C-6), 146.5 (C-10a), 152.3 (C-9), 158.3 (C-4a). HRMS-ESI [m/z]: Calculated for $C_{21}H_{31}N_3$ $[M+H]^+$:360.2201; Determined: 360.2228.

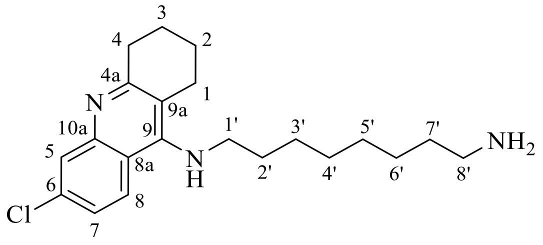

Figure S17. *N¹-(6-chloro-1,2,3,4-tetrahydroacridin-9-yl)octane-1,8-diamine (**6.l**).*

N^1-(6-chloro-1,2,3,4-tetrahydroacridin-9-yl)decane-1,10-diamine ***6.m***. Brown oil, yield 51 %. ^{1}H NMR (300 MHz, CD_3OD): δ 1.20-1.39 (12H, m, H-2′,4′,5′,6′,7′,9′), 1.60 (4H, p, *J* = 7.1 Hz, H-3′,8′), 1.86-1.94 (4H, m, H-2,3), 2.70 (2H, broad s, H-1), 2.85 (2H, t, *J* = 7.2 Hz, H-10′), 2.96 (2H, broad s, H-4), 3.55 (2H, t, *J* = 7.1 Hz, H-1′), 7.30 (1H, dd, *J* = 9.1 and 2.2 Hz, H-7), 7.73 (1H, d, *J* = 2.2 Hz, H-5), 8.08 (1H, d, *J* = 9.1 Hz, H-8). ^{13}C NMR (75 MHz, CD_3OD): δ 22.2 (C-2), 22.6 (C-3), 24.6 (C-1), 26.2 (C-9′), 26.4 (C-9′), 28.2 (C-8′), 28.9 (C-5′,6′), 29.0 (C-7′), 29.1 (C-4′), 30.8 (C-3′), 32.8 (C-4), 39.7 (C-10′), 48.2 (C-1′), 115.2 (C-9a), 118.0 (C-8a), 123.6 (C-7), 125.0 (C-5), 125.2 (C-8), 134.1 (C-6), 147.0 (C-10a), 152.0 (C-9), 158.7 (C-4a). HRMS-ESI [m/z]: Calculated for $C_{23}H_{35}ClN_3$ $[M+H]^+$: 388.2514; Determined: 388.2530.

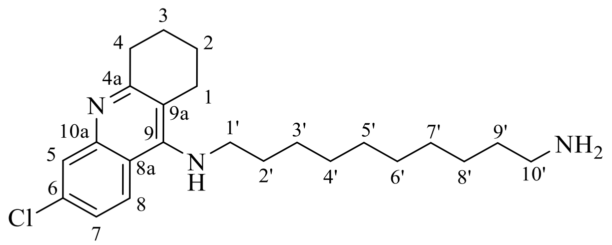

Figure S18. *N^1-(6-chloro-1,2,3,4-tetrahydroacridin-9-yl)decane-1,10-diamine (**6.m**).*

N^1-(6-chloro-1,2,3,4-tetrahydroacridin-9-yl)dodecane-1,12-diamine ***6.n***. Brown oil, ^{1}H NMR (300 MHz, CD_3OD): δ 1.19-1.43 (16H, m, H- 2',4',5',6',7',8',9',11'), 1.56-1.71 (4H, m, H-3′,10′), 1.89-1.96 (4H, m, H-2,3), 2.72 (2H, broad s, H-1), 2.89 (2H, t, *J* = 7.2 Hz, H-12′), 2.97 (2H, broad s, H-4), 3.59 (2H, t, *J* = 7.1 Hz, H-1′), 7.34 (1H, dd, *J* = 9.1 and 2.2 Hz, H-7), 7.75 (1H, d, *J* = 2.2 Hz, H-5), 8.12 (1H, d, *J* = 9.1 Hz, H-8). ^{13}C NMR (75 MHz, CD_3OD): δ 22.1 (C-2), 22.5 (C-3), 24.6 (C-1), 26.1 (C-11′), 26.4 (C-2′), 27.7 (C-10′), 28.9 (C-5′,8′), 29.1 (C-7′), 29.2 (C-6′), 30.7 (C-3′), 32.5 (C-4), 39.5 (C-12′), 48.2 (C-1′), 115.1 (C-9a), 117.8 (C-8a), 123.7 (C-7), 124.7 (C-5), 125.3 (C-8), 134.4 (C-6), 146.6 (C-10a), 152.3 (C-9), 158.4 (C-4a). HRMS-ESI [m/z]: Calculated for $C_{25}H_{39}ClN_3$ $[M+H]^+$: 416.2827; Determined: 416.2841.

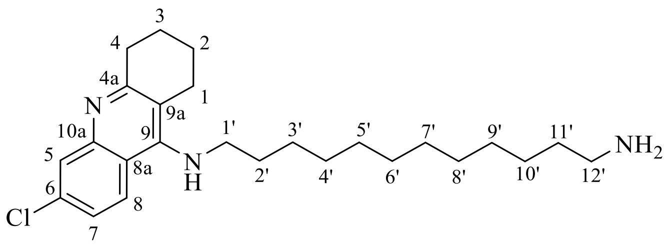

Figure S19. *N^1-(6-chloro-1,2,3,4-tetrahydroacridin-9-yl)dodecane-1,12-diamine (**6.n**).*

N^1,N^2-bis(1,2,3,4-tetrahydroacridin-9-yl)ethane-1,2-diamine ***10.a***, m.p. 120-125 ˚C (123-125 ˚C)[16]. ^{1}H NMR (300 MHz, CD_3OD): δ 1.61-1.71 (4H, m, H-3), 1.72-1.83 (4H, m, H-2), 2.30 (4H, t, *J* = 6.2 Hz, H-1), 2.80 (4H, t, *J* = 6.5 Hz, H-4), 3.97 (4H, s, H-1′), 7.28 (2H, ddd, *J* = 8.4, 6.5 and 1.5 Hz, H-7), 7.60 (2H, ddd, *J* = 8.4, 6.6 and 1.2 Hz, H-6), 7.66 (2H, dd, *J* = 8.4 and 1.4 Hz, H-5), 7.87 (2H, d, *J* = 8.4 Hz, H-8). ^{13}C NMR (75 MHz, CD_3OD): δ 21.5 (C-2), 22.1 (C-3), 24.6 (C-1), 31.1 (C-4), 48.4 (C-1′), 114.2 (C-9a), 118.2 (C-8a), 122.7 (C-8), 123.7 (C-7), 124.2 (C-5), 129.5 (C-6), 143.6 (C-10a), 152.5 (C-9), 155.4 (C-4a). HRMS-ESI [m/z]: Calculated for $C_{28}H_{31}N_4$ $[M+H]^+$: 423.2543; Determined: 423.2576.

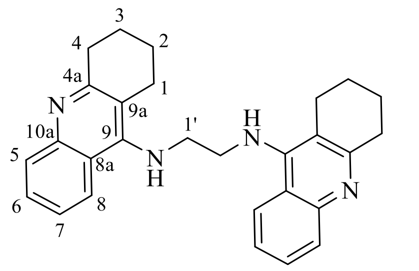

Figure S20. *N^1,N^2-bis(1,2,3,4-tetrahydroacridin-9-yl)ethane-1,2-diamine (**10.a**).*

N^1,N^3-bis(1,2,3,4-tetrahydroacridin-9-yl)propane-1,3-diamine ***10.b***. Yellow solid, m.p. 89-95 °C. ^{1}H NMR (300 MHz, $CDCl_3$): δ 1.78-1.93 (8H, m, H-2,3), 2.02 (2H, p, J = 6.8 Hz, H-2′), 2.61 (4H, t, J = 6.1 Hz, H-1), 3.04 (4H, t, J = 6.1 Hz, H-4), 3.60 (4H, t, J = 6.8 Hz, H-1′), 7.31 (2H, ddd, J = 8.3, 6.7 and 1.3 Hz, H-7), 7.55 (2H, ddd, J = 8.3; 6.7 and 1.3 Hz, H-6), 7.87 (2H, dd, J = 8.6 and 1.3 Hz, H-5), 7.91 (2H, dd, J = 8.6 and 1.3 Hz, H-8). ^{13}C NMR (75 MHz, $CDCl_3$): δ 22.7 (C-2), 22.9 (C-3), 24.9 (C-1), 33.3 (C-2′), 33.9 (C-4), 46.9 (C-1′), 116.6 (C-9a), 120.2 (C-8a), 122.4 (C-5), 124.0 (C-7), 128.5 (C-6), 128.7 (C-8), 147.3 (C-10a), 150.3 (C-9), 158.5 (C-4a). HRMS-ESI [m/z]: Calculated for $C_{29}H_{33}N_4$ $[M+H]^+$: 437.2700; Determined 437.2689.

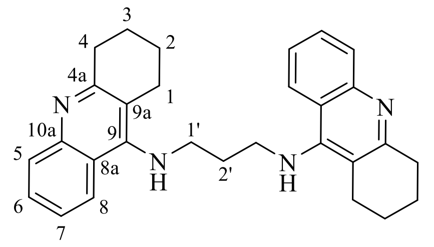

Figure S21. *N^1,N^3-bis(1,2,3,4-tetrahydroacridin-9-yl)propane-1,3-diamine (**10.b**).*

N^1,N^4-bis(1,2,3,4-tetrahydroacridin-9-yl)butane-1,4-diamine ***10.c***. Pale brown solid, m.p. 117-120 °C. ^{1}H NMR (300 MHz, CD_3OD): δ 1.67 (4H, broad s, H-2′), 1.75-1.88 (8H, m, H-2,3), 2.57 (4H, t, J = 5.9 Hz, H-1), 2.92 (4H, t, J = 6.1 Hz, H-4), 3.55 (4H, t, J = 6.2 Hz, H-1′), 7.29 (2H, ddd, J = 8.5, 6.8 and 1.3 Hz, H-7), 7.55 (2H, ddd, J = 8.5, 6.8 and 1.3 Hz, H-6), 7.74 (2H, dd, J = 8.5 and 1.1 Hz, H-5), 8.01 (2H, dd, J = 8.5 and 1.3 Hz, H-8). ^{13}C NMR (75 MHz, CD_3OD): δ 22.0 (C-2), 22.5 (C-3), 24.6 (C-1), 27.9 (C-2′), 32.2 (C-4), 47.5 (C-1′), 114.9 (C-9a), 119.3 (C-8a), 123.0 (C-8), 123.5 (C-7), 125.7 (C-5), 128.8 (C-6)145.4 (C-10a), 151.9 (C-9), 156.7 (C-4a). HRMS-ESI [m/z]: Calculated for $C_{30}H_{35}N_4$ $[M+H]^+$: 451.2856; Determined 451.2848.

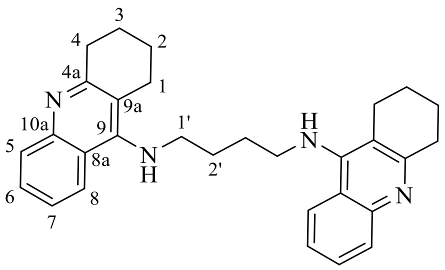

Figure S22. *N^1,N^4-bis(1,2,3,4-tetrahydroacridin-9-yl)butane-1,4-diamine (**10.c**).*

N^1,N^5-bis(1,2,3,4-tetrahydroacridin-9-yl)pentane-1,5-diamine ***10.d***. Brown oil. ^{1}H NMR (300 MHz, CD_3OD): δ 1.33-1.47 (2H, m, H-3′), 1.62 (4H, p, *J* = 7.6 Hz, H-2′,4′), 1.79-1.94 (8H, m, H-2,3), 2.67 (4H, t, *J* = 5.8 Hz, H-1), 2.95 (4H, t, *J* = 6.0 Hz, H-4), 3.48 (4H, t, *J* = 7.1 Hz, H-1′,5′), 7.32 (2H, ddd, *J* = 8.4, 6.8 and 1.3 Hz, H-7), 7.54 (2H, ddd, *J* = 8.4, 6.8 and 1.3 Hz, H-6), 7.76 (2H, dd, *J* = 8.4 and 1.3 Hz, H-5), 8.04 (2H, dd, *J* = 8.4 and 1.3 Hz, H-8). ^{13}C NMR (75 MHz, CD_3OD): δ 22.2 (C-3), 22.7 (C-2), 23.8 (C-3′), 24.7 (C-1), 30.5 (C-2′,4′), 32.6 (C-4), 48.1 (C-1′,5′), 115.3 (C-9a), 119.8 (C-8a), 123.0 (C-8), 123.4 (C-7), 126.3 (C-5), 128.5 (C-6), 146.2 (C-10a), 151.9 (C-9), 157.5 (C-4a). HRMS-ESI [m/z]: Calculated for $C_{31}H_{37}N_4$ $[M+H]^+$: 465.3013; Determined: 465.3030.

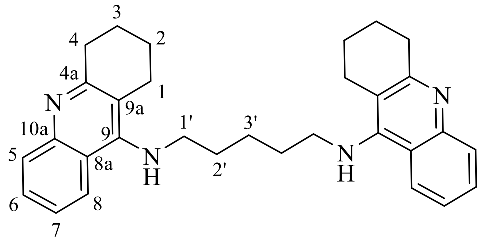

Figure S23. *N^1,N^5-bis(1,2,3,4-tetrahydroacridin-9-yl)pentane-1,5-diamine (**10.d**).*

N^1,N^8-bis(1,2,3,4-tetrahydroacridin-9-yl)octane-1,8-diamine ***10.e***. Ambar oil. ^{1}H NMR (300 MHz, $CDCl_3$): δ 1.27-1.42 (8H, m, H-3′,4′,5′,6′), 1.66 (4H, p, *J* = 7.1 Hz, H-2′,7′), 1.90 (4H, p, *J* = 3.1 Hz, H-2,3), 2.65-2.71 (4H, m, H-1), 3.03-3.13 (4H, t, *J* = 6.1 Hz, H-4), 3.52 (2H, t, *J* = 7.2 Hz, H-1′,8′), 7.34 (1H, dd, *J* = 8.5, 6.8 and 1.3 Hz, H-7), 7.42 (1H, dd, *J* = 8.5, 6.9 and 1.3 Hz, H-6), 7.55 (1H, dd, *J* = 8.5 and 1.3 Hz, H-5), 7.97 (1H, dd, *J* = 8.5 and 1.3 Hz, H-8).^{13}C NMR (75 MHz, $CDCl_3$): δ 22.5 (C-2), 22.9 (C-3), 24.7 (C-1), 26.8-29.2 (C-3′,4′,5′,6′), 31.7 (C-2′,7′), 33.3 (C-4), 49.3 (C-1′,8′), 115.2 (C-9a), 119.7 (C-8a), 123.0 (C-8), 123.7 (C-7), 127.7 (C-5), 128.7 (C-6), 146.4 (C-10a), 151.3 (C-9), 157.5 (C-4a). HRMS-ESI [m/z]: Calculated for $C_{34}H_{43}N_4$ $[M+H]^+$: 507.3482; Determined: 507.3561.

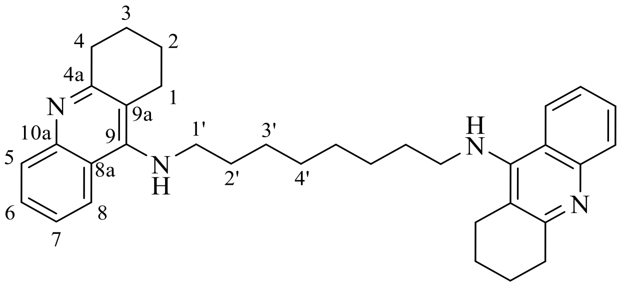

Figure S24. *N[1],N[8]-bis(1,2,3,4-tetrahydroacridin-9-yl)octane-1,8-diamine (**10.e**).*

N[1],N[10]-bis(1,2,3,4-tetrahydroacridin-9-yl)decane-1,10-diamine ***10.f***. Ambar oil. ^{1}H NMR (300 MHz, $(CD_3)_2CO$): δ 1.17-1.41 (16H, m, H-3',4',5',6',7',8'), 1.64 (8H, p, *J* = 7.1 Hz, H-2′,9′), 1.87 (8H, p, *J* = 3.2 Hz, H-2,3), 2.74-2.84 (4H, m, H-1), 2.93-3.00 (4H, m, H-4), 3.48 (4H, q, *J* = 6.7 Hz, H-1′,8′), 4.82 (2H, broad s, NH), 7.34 (2H, ddd, *J* = 8.4, 6.7 and 1.4 Hz, H-7), 7.52 (2H, ddd, *J* = 8.4, 6.8 and 1.4 Hz, H-6), 7.81 (2H, dd, *J* = 8.4 and 1.2 Hz, H-5), 8.12 (2H, dd, *J* = 8.4 and 1.3 Hz, H-8). ^{13}C NMR (75 MHz, $(CD_3)_2CO$): δ 22.8 (C-2), 23.1 (C-3), 25.0 (C-1), 26.7 (C-3′,8′), 28.7-29.5 (C-4′,5′,6′,7′), 31.1 (C-2′,9′), 33.9 (C-4), 48.8 (C-1′,10′), 116.2 (C-9a), 120.6 (C-8a), 123.0 (C-8), 123.2 (C-7), 127.7 (C-6), 128.8 (C-5), 147.7 (C-10a), 150.7 (C-9), 158.1 (C-4a). HRMS-ESI [m/z]: Calculated for $C_{36}H_{47}N_4$ $[M+H]^+$: 535.3795; Determined: 535.3809.

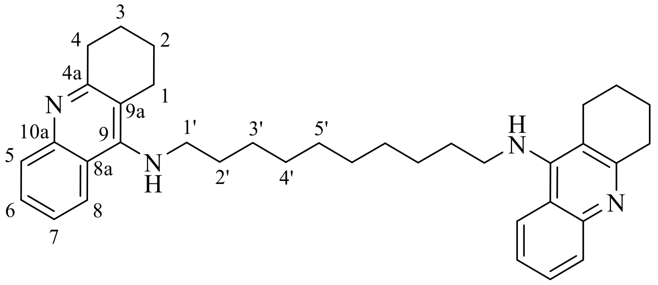

Figure S25. *N[1],N[10]-bis(1,2,3,4-tetrahydroacridin-9-yl)decane-1,10-diamine (**10.f**).*

N[1],N[12]-bis(1,2,3,4-tetrahydroacridin-9-yl)dodecane-1,12-diamine ***10.g***. Ambar oil. ^{1}H NMR (300 MHz, CD_3OD): δ 1.13-1.30 (12H, m, H-4′,5′,6′,7′,8′,9′), 1.24-1.39 (4H, m, H-2′,11′), 1.62 (4H, p, *J* = 7.9 and 7.1 Hz, H-3′,10′), 1.91 (8H, p; *J* = 3.1 Hz, H-2,3), 2.75 (4H, broad s, H-4), 2.97 (4H, broad s, H-1), 3.54 (4H, t, *J* = 7.2 Hz, H-1′,12′), 7.36 (2H, ddd, *J* = 8.4, 6.8 and 1.3 Hz, H-7), 7.55 (2H, ddd, *J* = 8.4, 6.8 and 1.4 Hz, H-6), 7.77 (2H, dd, *J* = 8.4 and 1.2 Hz, H-5), 8.10 (2H, dd, *J* = 8.4 and 1.3 Hz, H-8). ^{13}C NMR (75 MHz, CD_3OD): δ 22.3 (C-3), 22.7 (C-2), 24.7 (C-4), 26.4 (C-2′,11′), 28.9 (C-4′,9′), 29.0 (C-5′,8′), 29.1 (C-6′,7′), 30.8 (C-3′,10′), 32.6 (C-1), 48.3 (C-1′,12′), 115.2 (C-9a), 119.8 (C-8a), 123.1 (C-8), 123.3 (C-7), 126.3 (C-5), 128.4 (C-6), 146.3 (C-10a), 152.0 (C-9), 157.4 (C-4a). HRMS-ESI [m/z]: Calculated for $C_{38}H_{51}N_4$ $[M+H]^+$: 563.4108; Determined: 563.4095.

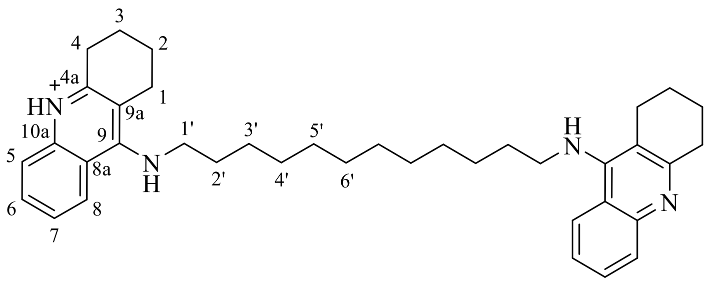

Figure S26. *N^1,N^{12}-bis(1,2,3,4-tetrahydroacridin-9-yl)dodecane-1,12-diamine (**10.g**).*

N^1,N^2-bis(6-chloro-1,2,3,4-tetrahydroacridin-9-yl)ethane-1,2-diamine ***10.h****,* White solid, m.p. 176-178 °C. ^{1}H NMR (500 MHz, CD_3OD): δ 1.63-1.70 (4H, m, H-2), 1.74-1.81 (4H, m, H-3), 2.29 (4H, t, J = 6.4 Hz, H-4), 2.82 (4H, t, J = 6.5 Hz, H-1), 3.88 (4H, s, H-1′), 7.16 (2H, dd, J = 9.1 and 2.2 Hz, H-7), 7.65 (2H, d, J = 2.2 Hz, H-5), 7.77 (2H, d, J = 9.1 Hz, H-8). ^{13}C NMR (125 MHz, (CD_3OD): δ 23.4 (C-3), 23.7 (C-2), 26.2 (C-4), 34.0 (C-1), 50.3 (C-1′), 116.4 (C-9a), 118.8 (C-8a), 124.9 (C-7), 125.8 (C-5), 126.5 (C-8), 135.4 (C-6), 148.1 (C-10a), 152.5 (C-9), 160.0 (C-4a). HRMS-ESI [m/z]: Calculated for $C_{28}H_{29}Cl_2N_4$ $[M+H]^+$: 491.1764; Determined: 491.1750.

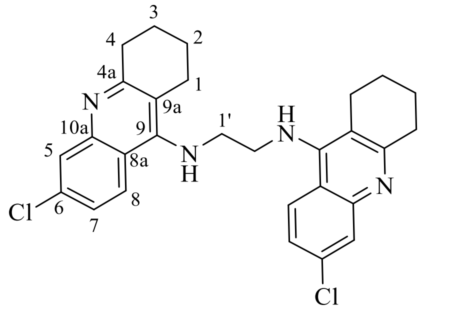

Figure S27. *N^1,N^2-bis(6-chloro-1,2,3,4-tetrahydroacridin-9-yl)ethane-1,2-diamine (**10.h**).*

N^1,N^3-bis(6-chloro-1,2,3,4-tetrahydroacridin-9-yl)propane-1,3-diamine ***10.i****.* Brown solid, m.p. 89-95 °C. ^{1}H NMR (300 MHz, CD_3OD): δ 1.68-1.84 (8H, m, H-2,3), 2.07 (2H, p, J = 5.9 Hz, H-2′), 2.43 (4H, t, J = 5.8 Hz, H-4), 2.78 (4H, t, J = 6.0 Hz, H-1), 3.69 (4H, t, J = 5.9 Hz, H-1′,3′), 7.17 (2H, dd, J = 9.1 and 2.2 Hz, H-7), 7.63 (2H, d, J = 2.2 Hz, H-5), 7.85 (2H, d, J = 9.1 Hz, H-8). ^{13}C NMR (75 MHz, CD_3OD): δ 21.8 (C-3), 22.2 (C-2), 24.4 (C-4), 32.0 (C-1), 32.6 (C-2′), 44.9 (C-1′), 114.2 (C-9a), 116.8 (C-8a), 123.4 (C-7), 124.7 (C-5), 125.2 (C-8), 134.2 (C-6), 146.1 (C-10a), 152.1 (C-9), 157.5 (C-4a). HRMS-ESI [m/z]: Calculated for $C_{29}H_{31}Cl_2N_4$ $[M+H]^+$: 505.1920; Determined: 505.1898.

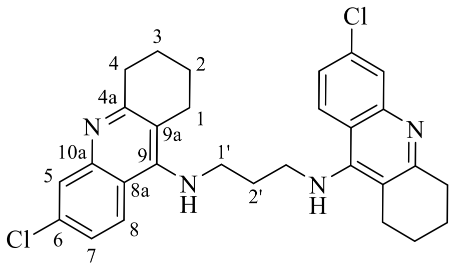

Figure S28. *N^1,N^3-bis(6-chloro-1,2,3,4-tetrahydroacridin-9-yl)propane-1,3-diamine (**10.i**)*

N^1,N^4-bis(6-chloro-1,2,3,4-tetrahydroacridin-9-yl)butane-1,4-diamine ***10.j***. White solid, m.p. 170-178 °C. ^{1}H NMR (300 MHz, DMSO-d$_6$): δ 1.54 (4H, broad s, H-2′), 1.66-1.82 (8H, m, H-2,3), 2.53 (4H, t, J = 6.0 Hz, H-1), 2.85 (4H, t, J = 6.0 Hz, H-4), 3.42 (4H, broad s, H-1), 5.88 (2H, broad s, NH), 7.27 (2H, dd, J = 9.1 and 2.3 Hz, H-7), 7.69 (2H, d, J = 2.3 Hz, H-5), 8.07 (2H, d, J = 9.1 Hz, H-8). ^{13}C NMR (75.47 MHz, (DMSO-d$_6$): δ 22.4 (C-2), 22.8 (C-3), 25.3 (C-1), 28.0 (C-2′), 33.1 (C-4), 47.6 (C-1′), 115.6 (C-9a), 118.3 (C8a), 124.0 (C-7), 126.0 (C-5; 8), 133.5 (C-6), 146.7 (C-10a), 151.3 (C-9), 158.5 (C-4a). HRMS-ESI [m/z]: Calculated for $C_{30}H_{33}Cl_2N_4$ $[M+H]^+$: 519.2077; Determined 519.2064.

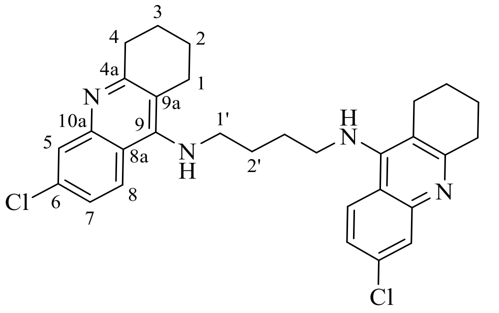

Figure S29. *N^1,N^4-bis(6-chloro-1,2,3,4-tetrahydroacridin-9-yl)butane-1,4-diamine (**10.j**).*

N^1,N^5-bis(6-chloro-1,2,3,4-tetrahydroacridin-9-yl)pentane-1,5-diamine ***10.k***. Brown oil. ^{1}H NMR (300 MHz, CDCl$_3$): δ 1.44-1.55 (2H, m, H-3′), 1.69 (4H, p, J = 7.3 Hz, H-2′,4′), 1.90 (8H, p, J = 3.4 Hz, H-2,3), 2.60-2.69 (4H, m, H-4), 2.98-3.08 (4H, m, H-1), 3.48 (4H, t, J = 7.2 Hz, H-1′,5′), 7.27 (2H, dd, J = 9.1 and 2.2 Hz, H-7), 7.86 (2H, d, J = 9.1 Hz, H-8), 7.90 (2H, d, J = 2.2 Hz, H-5). ^{13}C NMR (75 MHz, CDCl$_3$): δ 22.6 (C-3), 22.9 (C-2), 24.3 (C-4), 24.6 (C-3′), 31.5 (C-2′,4′), 33.9 (C-1), 49.3 (C-1′,5′), 116.0 (C-9a), 118.4 (C-8a), 124.4 (C-7), 124.4 (C-8), 127.45 (C-5), 134.2 (C-6), 147.9 (C-10a), 150.7 (C-9), 159.5 (C-4a). HRMS-ESI [m/z]: Calculated for $C_{32}H_{35}Cl_2N_4$ $[M+H]^+$: 533.2233; Determined 533.2225.

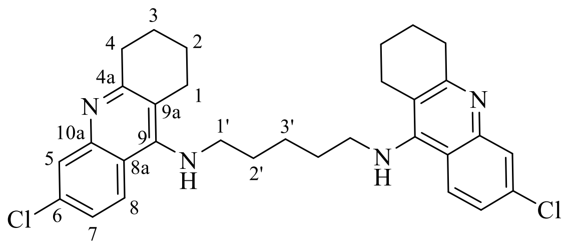

Figure S30. *N^1,N^5-bis(6-chloro-1,2,3,4-tetrahydroacridin-9-yl)pentane-1,5-diamine (**10.k**).*

N^1,N^8-bis(6-chloro-1,2,3,4-tetrahydroacridin-9-yl)octane-1,8-diamine **10.l**. Brown oil. ^{1}H NMR (300 MHz, CD_3OD): δ 1.21-1.35 (8H, m, H-2′,4′,5′,7′), 1.61 (4H, p, J = 7.1 Hz, H-3′,6′), 1.86-1.94 (4H, m, H-2,3), 2.64-2.72 (2H, m, H-1), 2.91-2.99 (2H, m, H-4), 3.55 (4H, t, J = 7.2 Hz, H-1′,8′), 7.30 (2H, dd, J = 9.1 and 2.2 Hz, H-7), 7.71 (2H, d, J = 2.2 Hz, H-5), 8.08 (2H, d, J = 9.1 Hz, H-8). ^{13}C NMR (75 MHz, CD_3OD): δ 23.4 (C-3), 23.8 (C-2), 25.9 (C-4), 27.6 (C-4′,5′), 30.1 (C-2′,7′), 32.0 (C-3′,6′), 33.7 (C-1), 48.9 (C-1′,8′), 116.3 (C-9a), 119.0 (C-8a), 125.1 (C-7), 125.8 (C-8), 126.8 (C-5), 136.0 (C-6), 147.7 (C-10a), 153.8 (C-9), 159.4 (C-4a). HRMS-ESI [m/z]: Calculated for $C_{34}H_{41}Cl_2N_4$ $[M+H]^+$: 575.2703; Determined 575.2692.

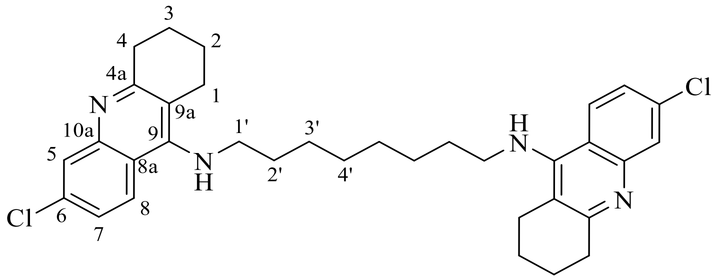

Figure S31. *N^1,N^8-bis(6-chloro-1,2,3,4-tetrahydroacridin-9-yl)octane-1,8-diamine (**10.l**).*

N^1,N^{10}-bis(6-chloro-1,2,3,4-tetrahydroacridin-9-yl)decane-1,10-diamine **10.m**. Brown oil. ^{1}H NMR (300 MHz, CD^3OD): δ 1.14-1.37 (8H, m, H-4′,5′,6′,7′), 1.62 (4H, p, J = 7.1 Hz, H-3′,8′), 1.91 (8H, p, J = 3.7 Hz, H-2,3), 2.68-2.76 (4H, m, H-4), 2.92-3.00 (4H, m, H-1), 3.56 (4H, t, J = 7.1 Hz, H-1′,10′), 7.31 (2H, dd, J = 9.1 and 2.2 Hz, H-7), 7.74 (2H, d, J = 2.2 Hz, H-5), 8.09 (2H, d, J = 9.1 Hz, H-8). ^{13}C NMR (75 MHz, CD^3OD): δ 22.1 (C-3), 22.6 (C-2), 24.6 (C-4), 26.3 (C-2′,9′), 28.7-28.8 (C-4′,5′,6′,7′), 30.7 (C-3′,8′), 32.7 (C-1), 48.2 (C-1′,10′), 115.2 (C-9a), 118.0 (C-8a), 123.6 (C-7), 125.0 (C-5), 125.2 (C-8), 134.2 (C-6), 147.0 (C-10a), 152.1 (C-9), 158.7 (C-4a). HRMS-ESI [m/z]: Calculated for $C^{36}H^{45}Cl^{2}N^{4}$ $[M+H]^+$: 603,3016; Determined: 603.3014.

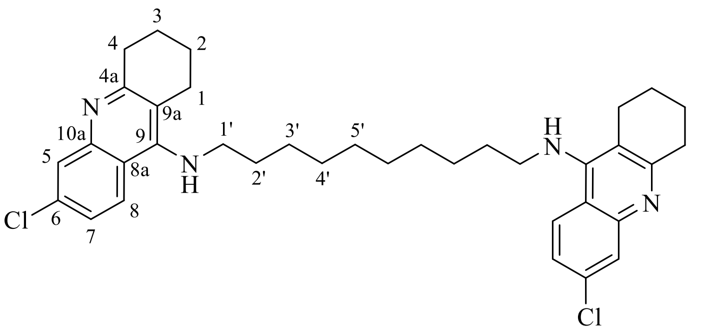

Figure S32. *N^1,N^{10}-bis(6-chloro-1,2,3,4-tetrahydroacridin-9-yl)decane-1,10-diamine (**10.m**).*

N^1,N^{12}-bis(6-chloro-1,2,3,4-tetrahydroacridin-9-yl)dodecane-1,12-diamine ***10.n***. Brown oil. ^{1}H NMR (500 MHz, CD_3OD): *δ* 1.23-1.29 (4H, m, H-4′,9′), 1.30-1.39 (4H, m, H-2′,11′), 1.65 (4H, p, *J* = 7.2 Hz, H-3′,10′), 1.93 (8H, p, *J* = 3.3 Hz, H-2,3), 2.71-2.76 (4H, m, H-4), 2.95-3.00 (4H, m, H-1), 3.58 (4H, t, *J* = 7.1 Hz, H-1′,12′), 7.33 (2H, dd, *J* = 9.1 and 2.2 Hz, H-7), 7.75 (2H, d, *J* = 2.2 Hz, H-5), 8.11 (2H, d, *J* = 9.1 Hz, H-8). ^{13}C NMR (125 MHz, CD_3OD): *δ* 22.2 (C-3), 22.6 (C-2), 24.6 (C-4), 26.3 (C-2′,11′), 28.8 (C-4′,9′), 29.0 (C-5′,8′), 29.0 (C-6′,7′), 30.8 (C-3′,10′), 32.8 (C-1), 48.2 (C-1′,12′), 115.3 (C-9a), 118.0 (C-8a), 123.6 (C-7), 125.1 (C-5), 125.2 (C-8), 134.2 (C-6), 147.1 (C-10a), 152.1 (C-9), 158.8 (C-4a). HRMS-ESI [m/z]: Calculated for $C_{38}H_{49}Cl_2N_4$ $[M+H]^+$: 631.3329; Determined: 631.3345.

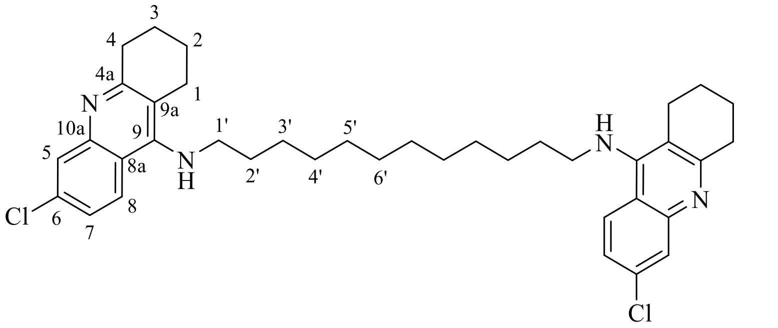

Figure S33. *N^1,N^{12}-bis(6-chloro-1,2,3,4-tetrahydroacridin-9-yl)dodecane-1,12-diamine (**10.n**).*

General procedure for the synthesis of *N*-acetylated-9-alkylamino-1,2,3,4-tetrahydroacridines (7.a-n):

Derivative **7.a as example of NMR spectra.*

N-{2-[(1,2,3,4-tetrahydroacridin-9-yl)amino]ethyl}acetamide ***7.a***, Yellow oil. ^{1}H NMR (300 MHz, CD_3OD): *δ* 1.88-1.97 (4H, m, H-2,3), 1.93 (3H, s, CH_3), 2.75 (2H, broad s, H-1), 2.99 (2H, broad s, H-4), 3.46 (2H, t, *J* = 6.1 Hz, H-2'), 3.73 (2H, t, *J* = 6.1 Hz, H-1'), 7.41 (1H, ddd, *J* = 8.4, 6.8 and 1.3 Hz, H-7), 7.60 (1H, ddd, *J* = 8.4, 6.8 and 1.3 Hz, H-6), 7.77 (1H, dd, *J* = 8.4 and 1.3 Hz, H-5), 8.15 (1H, dd, *J* = 8.4 and 1.3 Hz, H-8). ^{13}C NMR (75 MHz, CD_3OD): *δ* 21.1 (CH_3-amide), 22.0 (C-2), 22.5 (C-3), 24.6 (C-1), 32.1 (C-4), 39.9 (C-2'), 48.3 (C-1'), 105.0 (C-9a), 119.2 (C-8a), 123.2 (C-8), 123.7 (C-7), 125.4 (C-5), 129.0 (C-6), 145.2 (C-10a), 152.3 (C-9), 156.7 (C-4a), 172.9 (C=O). HRMS-ESI [m/z]: Calculated for $C_{17}H_{22}N_3O$ $[M+H]^+$: 284.1757; Determined: 284.1768.

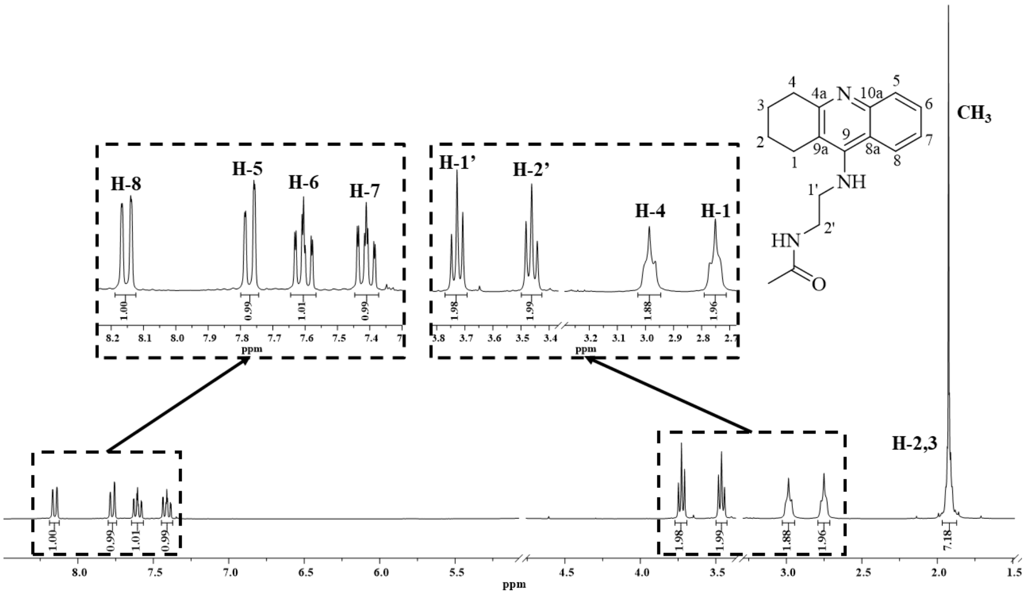

Figure S34. *^{1}H NMR N-{2-[(1,2,3,4-tetrahydroacridin-9-yl)amino]ethyl}acetamide (**7.a**).*

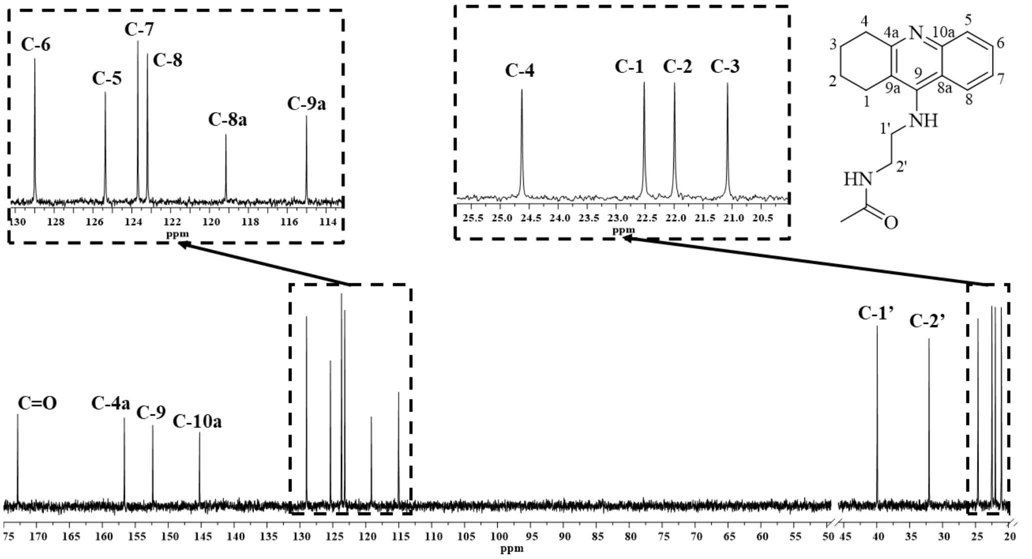

Figure S35. *^{13}C NMR N-{2-[(1,2,3,4-tetrahydroacridin-9-yl)amino]ethyl}acetamide (**7.a**).*

N-{3-[(1,2,3,4-tetrahydroacridin-9-yl)amino]propyl}acetamide ***7.b***. Ambar oil. ^{1}H NMR (500 MHz, CD_3OD): δ 1.95 (3H, s, CH_3), 1.96-2.03 (4H, m, H-2,3), 1.99-2.03 (2H, m, H-2′), 2.77 (2H, t, J = 5.8 Hz, H-1), 3.04 (2H, t, J = 5.9 Hz, H-4), 3.34 (2H, t, J = 6.5 Hz, H-3′), 3.97 (2H, t, J = 6.7 Hz, H-1′), 7.60 (1H, ddd, J = 8.5, 6.8 and 1.4 Hz, H-7), 7.79 (1H, dd, J = 8.5 and 1.4 Hz, H-5), 7.85 (1H, ddd, J = 8.5, 6.8 and 1.2 Hz, H-6), 8.39 (1H, dd, J = 8.5 and 1.1 Hz, H-8). ^{13}C NMR (125 MHz, CD_3OD): δ 20.5 (CH_3-amide), 21.1 (C-2), 21.7 (C-3), 23.6 (C-1), 28.1 (C-4), 30.2 (C-2′), 35.8 (C-3′), 44.6 (C-1′), 111.9 (C-9a), 115.9 (C-8a), 119.2 (C-5), 124.9 (C-7), 124.9 (C-8), 132.5 (C-6), 138.8 (C-10a), 150.8 (C-9), 156.5 (C-4a), 172.6 (C=O). HRMS-ESI [m/z]: Calculated for $C_{18}H_{24}N_3O$ $[M+H]^+$: 298.1914; Determined: 298.1919.

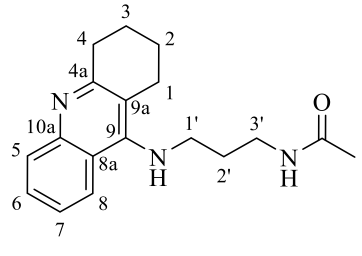

Figure S36. *N-{3-[(1,2,3,4-tetrahydroacridin-9-yl)amino]propyl}acetamide (**7.b**).*

N-{4-[(1,2,3,4-tetrahydroacridin-9-yl)amino]butyl}acetamide ***7.c***. Ambar oil. ^{1}H NMR (300 MHz, CD_3OD): δ 1.62 (2H, p, J = 6.9 Hz, H-2′), 1.85 (2H, p, J = 6.9 Hz, H-3′), 1.92 (3H, s, CH_3), 1.98 (4H, p, J = 3.1Hz, H-2,3), 2.69-2.77 (2H, m, H-4), 2.99-3.07 (2H, m, H-1), 3.21 (2H, t, J = 7.0 Hz, H-4′), 3.95 (2H, t, J = 7.2 Hz, H-1′), 7.59 (1H, ddd, J = 8.5, 6.5 and 1.3 Hz, H-7), 7.80 (1H, dd, J = 8.5 and 1.3 Hz, H-5), 7.83 (1H, ddd, J = 8.5, 6.5 and 1.3 Hz, H-6), 8.38 (1H, dd, J = 8.5 and 1.3 Hz, H-8). ^{13}C NMR (75 MHz, CD3OD): δ 20.6 (C-3), 21.2 (CH_3-amide), 21.7 (C-2), 23.6 (C-4), 26.2 (C-2′), 27.5 (C-3′),

28.4 (C-1), 38.5 (C-4′), 47.3 (C-1′), 112.0 (C-9a), 116.1 (C-8a), 119.5 (C-5), 124.8 (C-8), 124.9 (C-7), 132.3 (C-6), 139.2 (C-10a), 151.1 (C-4a), 156.1 (C-9), 172.0 (C=O). HRMS-ESI [m/z]: Calculated for $C_{19}H_{26}N_3O$ $[M+H]^+$: 312.2070; Determined: 312.2075.

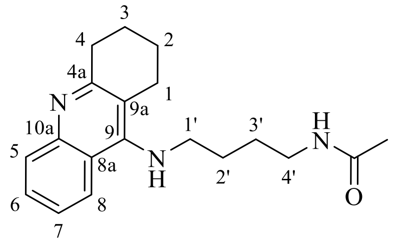

Figure S37. *N-{4-[(1,2,3,4-tetrahydroacridin-9-yl)amino]butyl}acetamide (**7.c**).*

N-{5-[(1,2,3,4-tetrahydroacridin-9-yl)amino]pentyl}acetamide ***7.d***. Pale brown solid, m.p. 235-238 °C. ^1H NMR (300 MHz, CD_3OD): δ 1.35-1.47 (2H, m, H-3′), 1.47-1.61 (2H, m, H-4′), 1.74 (2H, p, J = 7.5 Hz, H-2′), 1.87-2.00 (4H, m, H-2,3), 1.92 (3H, s, CH_3-amide), 2.74 (2H, broad s, H-4), 2.99 (2H, broad s, H-1), 3.16 (2H, t, J = 6.9 Hz, H-5′), 3.70 (2H, t, J = 7.2 Hz, H-1′), 7.45 (1H, ddd, J = 8.4, 6.8 and 1.3 Hz, H-7), 7.67 (1H, ddd, J = 8.4, 6.8 and 1.3 Hz, H-6), 7.78 (1H, dd, J = 8.4 and 1.3 Hz, H-5), 8.21 (1H, dd, J = 8.4 and 1.2 Hz, H-8). ^{13}C NMR (75 MHz, CD_3OD): δ 21.1 (CH_3-amide), 21.6 (C-3), 22.3 (C-2), 23.8 (C-3′), 24.3 (C-4), 28.7 (C-4′), 30.2 (C-2′), 30.9 (C-1), 38.8 (C-5′), 48.0 (C-1′), 113.9 (C-9a), 118.3 (C-8a), 123.6 (C-5), 123.8 (C-8), 123.9 (C-7), 130.0 (C-6), 143.4 (C-10a), 153.6 (C-9), 154.9 (C-4a), 171.8 (C=O). HRMS-ESI [m/z]: Calculated for $C_{20}H_{28}N_3O$ $[M+H]^+$: 326.2227; Determined: 326.2224.

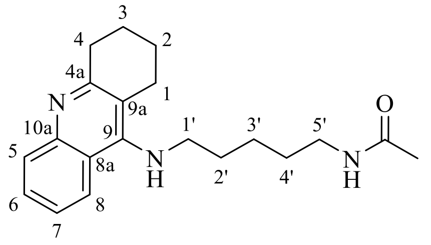

Figure S38. *N-{5-[(1,2,3,4-tetrahydroacridin-9-yl)amino]pentyl}acetamide (**7.d**).*

N-{8-[(1,2,3,4-tetrahydroacridin-9-yl)amino]octyl}acetamide ***8.e***. Brown oil. ^1H NMR (300 MHz, $CDCl_3$): δ 1.18-1.35 (8H, m, H-2′,4′,5′,7′), 1.44 (2H, t, J = 6.9 Hz, H-6′), 1.64 (2H, p, J = 7.1 Hz, H-3′), 1.90 (4H, dt, J = 6.3 and 2.9 Hz, H-2,3), 1.95 (3H, s, CH_3-amide), 2.69 (2H, broad s, H-4), 3.05 (2H, broad s, H-1), 3.10-3.28 (2H, m, H-8′), 3.49 (2H, t, J = 7.2 Hz, H-1′), 4.03 (1H, broad s, NH), 5.90 (1H, broad s, NH-amide), 7.33 (1H, ddd, J = 8.4, 6.8 and 1.3 Hz, H-7), 7.54 (1H, ddd, J = 8.4, 6.8 and 1.3 Hz, H-6), 7.91 (1H, d, J = 8.4 Hz, H-5), 7.96 (1H, d, J = 8.4 Hz, H-8). ^{13}C NMR (75 MHz, $CDCl_3$): δ 22.6 (C-2), 22.9 (C-3), 23.3 (CH_3-amide), 24.7 (C-4), 26.7 (C-2′), 26.7 (C-7′), 29.1 (C-5′), 29.1 (C-4′), 29.5 (C-6′), 31.7 (C-3′), 33.6 (C-1), 39.6 (C-8′), 49.4 (C-1′), 115.5 (C-9a), 119.9 (C-8a), 123.0 (C-8),

123.7 (C-7), 128.0 (C-5), 128.5 (C-6), 146.9 (C-10a), 151.2 (C-9), 157.9 (C-4a), 170.2 (C=O). HRMS-ESI [m/z]: Calculated for $C_{23}H_{34}N_3O$ [M+H]$^+$: 368.2696; Determined: 368.2681.

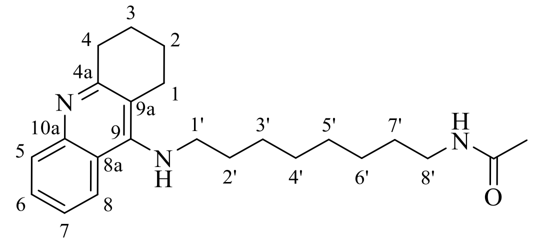

Figure S39. *N-{8-[(1,2,3,4-tetrahydroacridin-9-yl)amino]octyl}acetamide (**7.e**).*

N-{10-[(1,2,3,4-tetrahydroacridin-9-yl)amino]decyl}acetamide ***7.f***. White solid, m.p. 57-65 °C. ^{1}H NMR (300 MHz, CD_3OD): δ 1.25-1.41 (16H, m, H-2',4',5',6',7',9'), 1.48 (2H, p, *J* = 7.0 Hz, H-8′), 1.70 (2H, p, *J* = 7.5 Hz, H-3′), 1.91-1.96 (4H, m, H-2,3), 1.94 (3H, s, CH_3-amide), 2.69-2.77 (2H, m, H-4), 2.98-3.06 (2H, m, H-1), 3.14 (2H, t, *J* = 7.1 Hz, H-10′), 3.67 (2H, t, *J* = 7.2 Hz, H-1′), 7.44 (1H, ddd, *J* = 8.5, 6.8 and 1.3 Hz, H-7), 7.65 (1H, ddd, *J* = 8.5, 6.8 and 1.3 Hz, H-6), 7.82 (1H, dd, *J* = 8.5 and 1.3 Hz, H-5), 8.19 (1H, dd, *J* = 8.5 and 1.3 Hz, H-8). ^{13}C NMR (75 MHz, CD_3OD): δ 21.1 (CH_3-amide), 21.7 (C-3), 22.4 (C-2), 24.3 (C-4), 26.4 (C-2′), 26.6 (C-9′), 28.9 (C-8′), 28.9-29.1 (C-4′,5′,6′,7′), 30.6 (C-3′), 31.1 (C-1), 39.1 (C-10′), 48.1 (C-1′), 114.0 (C-9a), 118.5 (C-8a), 123.6 (C-8), 123.8 (C-7), 124.1 (C-5), 129.7 (C-6), 143.8 (C-4a), 153.4 (C-10a), 155.2 (C-9), 171.7 (C=O). HRMS-ESI [m/z]: Calculated for $C_{25}H_{38}N_3O$ [M+H]$^+$: 396.3009; Determined: 396.3000.

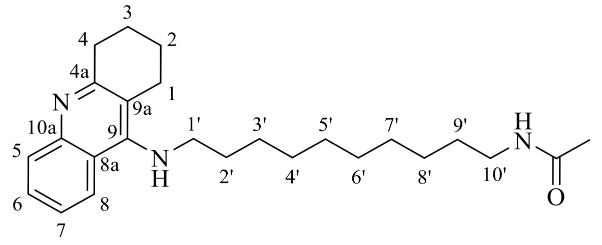

Figure S40. *N-{10-[(1,2,3,4-tetrahydroacridin-9-yl)amino]decyl}acetamide (**7.f**).*

N-{12-[(1,2,3,4-tetrahydroacridin-9-yl)amino]dodecyl}acetamide ***7.g***. White solid, 55-63 °C. ^{1}H NMR (300 MHz, CD_3OD): δ 1.21-1.40 (16H, m, H-2',4',5',6',7',8',9',11'), 1.48 (2H, p, *J* = 7.1 Hz, H-10′), 1.66 (2H, p, *J* = 7.2 Hz, H-3′), 1.94 (3H, s, CH_3-amide), 1.88-1.95 (4H, m, H-2,3), 2.70-2.77 (2H, m, H-4), 2.96-3.03 (2H, m, H-1), 3.14 (2H, t, *J* = 7.1 Hz, H-12′), 3.60 (2H, t, *J* = 7.2 Hz, H-1′), 7.40 (1H, ddd, *J* = 8.5, 6.8 and 1.3 Hz, H-7), 7.60 (1H, ddd, *J* = 8.5, 6.8 and 1.3 Hz, H-6), 7.79 (1H, dd, *J* = 8.5 and 1.3 Hz, H-5), 8.14 (1H, dd, *J* = 8.5 and 1.3 Hz, H-8). ^{13}C NMR (75 MHz, CD_3OD): δ 21.1 (CH_3-amide), 22.0 (C-3), 22.5 (C-2), 24.5 (C-4), 26.5 (C-2′), 26.6 (C-11′), 28.9 (C-10′), 29.0-29.3 (C-4′,5′,6′,7′,8′,9′), 30.8 (C-3′), 31.9 (C-1), 39.1 (C-12′), 48.2 (C-1′), 114.6 (C-9a), 119.1 (C-8a), 123.4 (C-8), 123.5 (C-7),

125.2 (C-5), 129.1 (C-6), 145.1 (C-10a), 152.7 (C-9), 156.3 (C-4a), 171.7 (C=O). HRMS-ESI [m/z]: Calculated for $C_{27}H_{42}N_3O$ $[M+H]^+$: 424.3322; Determined: 424.3327.

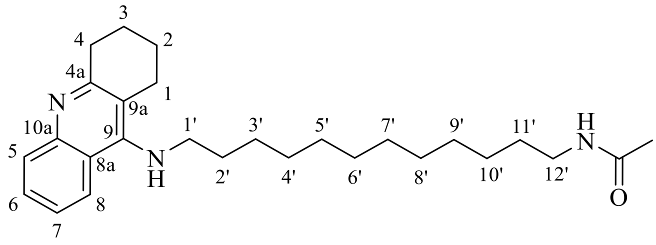

Figure S41. *N-{12-[(1,2,3,4-tetrahydroacridin-9-yl)amino]dodecyl}acetamide (**7.g**).*

N-{2-[(6-chloro-1,2,3,4-tetrahydroacridin-9-yl)amino]ethyl}acetamide ***7.h***, Amber oil. ^1H NMR (300 MHz, CD_3OD): δ 1.89-2.01 (4H, m, H-2,3), 1.95 (3H, s, CH_3-amide), 2.68-2.78 (2H, m, H-4), 2.93-3.03 (2H, m, H-1), 3.51 (2H, t, *J* = 5.9 Hz, H-2′), 3.85 (2H, t, *J* = 5.9 Hz, H-1′), 7.42 (1H, dd, *J* = 9.2 and 2.2 Hz, H-7), 7.75 (1H, d, *J* = 2.2 Hz, H-5), 8.25 (1H, d, *J* = 9.2 Hz, H-8). ^{13}C NMR (75 MHz, CD_3OD): δ 21.0 (CH_3-amide), 21.5 (C-3), 22.1 (C-2), 24.2 (C-4), 31.0 (C-1), 39.7 (C-2′), 48.6 (C-1′), 114.3 (C-9a), 116.5 (C-8a), 122.4 (C-5), 124.4 (C-7), 125.9 (C-8), 135.8 (C-6), 144.1 (C-10a), 153.6 (C-9), 155.9 (C-4a), 173.2 (C=O). HRMS-ESI [m/z]: Calculated for $C_{17}H_{21}ClN_3O$ $[M+H]^+$: 318.1368; Determined 318.1375.

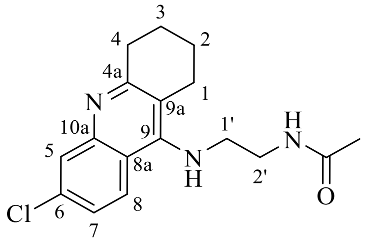

Figure S42. *N-{2-[(6-chloro-1,2,3,4-tetrahydroacridin-9-yl)amino]ethyl}acetamide (**7.h**).*

N-{3-[(6-chloro-1,2,3,4-tetrahydroacridin-9-yl)amino]propyl}acetamide ***7.i***. White solid, m.p. 120-125 °C. ^1H NMR (300 MHz, CD_3OD): δ 1.91 (2H, p, *J* = 6.7 Hz, H-2′), 1.93-1.96 (4H, m, H-2; 3), 1.96 (3H, s, CH_3), 2.74 (2H, broad s, H-1), 3.01 (2H, broad s, H-4), 3.31 (2H, t, *J* = 6.6 Hz, H-3′), 3.76 (2H, t, *J* = 6.7 Hz, H-1′), 7.43 (1H, dd, *J* = 9.2 and 2.2 Hz, H-7), 7.78 (1H, d, *J* = 2.2 Hz, H-5), 8.22 (1H, d, *J* = 9.2 Hz, H-8). ^{13}C NMR (75 MHz, CD_3OD): δ 21.1 (CH_3-amide), 21.3 (C-2), 22.0 (C-3), 24.1 (C-1), 30.3 (C-2′), 30.4 (C-4), 36.0 (C-3′), 44.8 (C-1′), 113.9 (C-9a), 116.1 (C-8a), 121.6 (C-5), 124.6 (C-7), 126.0 (C-8), 136.3 (C-6), 143.1 (C-10a), 154.1 (C-9), 155.0 (C-4a). HRMS-ESI [m/z]: Calculated for $C_{18}H_{23}ClN_3O$ $[M+H]^+$: 332.1524; Determined 332.1532.

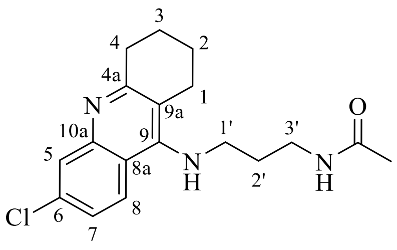

Figure S43. *N-{3-[(6-chloro-1,2,3,4-tetrahydroacridin-9-yl)amino]propyl}acetamide* (***7.i***).

N-{4-[(6-chloro-1,2,3,4-tetrahydroacridin-9-yl)amino]butyl}acetamide ***7.j***. Ambar oil. ^{1}H NMR (300 MHz, CD_3OD): δ 1.54-1.66 (2H, m, H-2′), 1.73-1.85 (2H, m, H-3′), 1.92 (3H, s, CH_3), 1.92-1.98 (4H, m, H-2,3), 2.68-2.75 (2H, m, H-4), 2.96-3.03 (2H, m, H-1), 3.20 (2H, t, J = 6.9 Hz, H-4′), 3.81 (2H, t, J = 7.2 Hz, H-1′), 7.46 (1H, dd, J = 9.2 and 2.2 Hz, H-7), 7.76 (1H, d, J = 2.2 Hz, H-5), 8.26 (1H, d, J = 9.2 Hz, H-8). ^{13}C NMR (75 MHz, CD_3OD): δ 21.1 (C-3), 21.2 (CH_3-amide), 21.9 (C-2), 23.9 (C-4), 26.2 (C-2′), 27.6 (C-3′), 30.0 (C-1), 38.5 (C-4′), 47.6 (C-1′), 113.5 (C-9a), 115.7 (C-8a), 120.8 (C-5), 124.7 (C-7), 126.4 (C-8), 136.8 (C-6), 142.4 (C-10a), 154.1 (C-4a), 154.5 (C-9), 171.9 (C=O). HRMS-ESI [m/z]: Calculated for $C_{20}H_{27}ClN_3O$ $[M+H]^+$: 346.1681; Determined 346.1670.

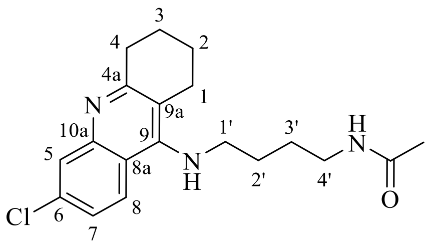

Figure S44. *N-{4-[(6-chloro-1,2,3,4-tetrahydroacridin-9-yl)amino]butyl}acetamide* (***7.j***).

N-{5-[(6-chloro-1,2,3,4-tetrahydroacridin-9-yl)amino]pentyl}acetamide ***7.k***. Ambar oil. ^{1}H NMR (300 MHz, CD_3OD): δ 1.36-1.48 (2H, m, H-3′), 1.48-1.62 (2H, m, H-4′), 1.68-1.85 (2H, m, H-2′), 1.91-1.97 (7H, m, H-2,3,CH_3), 2.71 (2H, broad s, H-1), 3.00 (2H, broad s, H-4), 3.17 (2H, t, J = 6.9 Hz, H-5′), 3.73 (2H, t, J = 7.2 Hz, H-1′), 7.42 (1H, dd, J = 9.2 and 2.2 Hz, H-7), 7.77 (1H, d, J = 2.2 Hz, H-5), 8.21 (1H, d, J = 9.2 Hz, H-8). ^{13}C NMR (75 MHz, CD_3OD): δ 21.1 (C-2), 21.4 (C-3), 22.1 (CH_3-amide), 23.7 (C-1), 24.1 (C-3′), 28.7 (C-4′), 30.1 (C-2′), 30.7 (C-4), 38.8 (C-5), 48.0 (C-1′), 113.9 (C-9a), 116.3 (C-8a), 122.1 (C-5), 124.4 (C-7), 126.0 (C-8), 136.0 (C-6), 143.7 (C-4a), 153.8 (C-10a), 155.4 (C-9), 171.8 (C=O). HRMS-ESI [m/z]: Calculated for $C_{22}H_{31}ClN_3O$ $[M+H]^+$: 360.1837; Determined 360.1812.

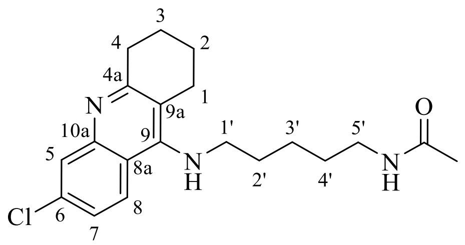

Figure S45. *N-{5-[(6-chloro-1,2,3,4-tetrahydroacridin-9-yl)amino]pentyl}acetamide (**7.k**).*

N-{8-[(6-chloro-1,2,3,4-tetrahydroacridin-9-yl)amino]octyl}acetamide ***7.l***. Brown oil. ^{1}H NMR (300 MHz, $CDCl_3$): δ 1.17-1.39 (8H, m, H-2′,4′,5′,7′), 1.44 (2H, t, J = 7.0 Hz, H-6′), 1.64 (2H, p, J = 7.1 Hz, H-3′), 1.89 (4H, p, J = 3.1 Hz, H-2,3), 1.96 (3H, s, CH_3-amide), 2.64 (2H, broad s, H-4), 3.03 (2H, broad s, H-1), 3.19 (2H, q, J = 6.7 Hz, H-8′), 3.49 (2H, t, J = 7.2 Hz, H-1′), 4.13 (1H, broad s, NH), 5.83 (1H, broad s, NH-amide), 7.25 (1H, dd, J = 9.3 and 2.2 Hz, H-7), 7.89 (1H, s, H-5), 7.90 (1H, d, J = 9.3 Hz, H-8). ^{13}C NMR (75 MHz, $CDCl_3$): δ 22.5 (C-3), 22.8 (C-2), 23.3 (CH_3-amide), 24.5 (C-4), 26.7 (C-2′; 7′), 29.1 (C-5′), 29.1 (C-4′), 29.5 (C-6′), 31.7 (C-3′), 33.6 (C-1), 39.6 (C-8′), 49.4 (C-1′), 115.3 (C-9a), 118.0 (C-8a), 124.3 (C-7), 124.8 (C-8), 126.8 (C-5), 134.3 (C-6), 147.4 (C-10a), 151.2 (C-9), 159.0 (C-4a), 170.2 (C=O). HRMS-ESI [m/z]: Calculated for $C_{25}H_{37}ClN_3O$ $[M+H]^+$: 402.2307; Determined 402.2310.

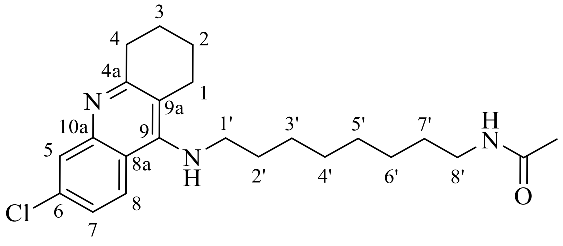

Figure S46. *N-{8-[(6-chloro-1,2,3,4-tetrahydroacridin-9-yl)amino]octyl}acetamide (**7.l**).*

N-{10-[(6-chloro-1,2,3,4-tetrahydroacridin-9-yl)amino]decyl}acetamide ***7.m***. Brown oil. ^{1}H NMR (300 MHz, CD_3OD): δ 1.23-1.39 (12H, m, H-2',4',5',6',7',9'), 1.48 (2H, p, J = 7.3 Hz, H-8′), 1.67 (2H, p, J = 7.4 Hz, H-3′), 1.90-1.96 (4H, m , H-2,3), 1.93 (3H, s, CH_3-amide), 2.69-2.76 (2H, m, H-4), 2.94-3.02 (2H, m, H-1), 3.14 (2H, t, J = 7.1 Hz, H-10′), 3.61 (2H, t, J = 7.2 Hz, H-1), 7.35 (1H, dd, J = 9.1 and 2.2 Hz, H-7), 7.75 (1H, d, J = 2.2 Hz, H-5), 8.14 (1H, d, J = 9.1 Hz, H-8). ^{13}C NMR (75 MHz, (CD_3OD): δ 21.1 (C-3), 22.0 (C-2), 22.5 (CH_3-amide), 24.5 (C-4), 26.4 (C-2′), 26.6 (C-9′), 28.9 (C-8′), 28.9-29.3 (C-4′,5′,6′,7′), 30.7 (C-3′), 32.3 (C-1), 39.1 (C-10′), 48.2 (C-1′), 114.9 (C-9a), 117.6 (C-8a), 123.8 (C-7), 124.4 (C-5), 125.4 (C-8), 134.6 (C-6), 148.3 (C-10a), 152.5 (C-9), 158.0 (C-4a), 171.7 (C=O). HRMS-ESI [m/z]: Calculated for $C_{27}H_{41}ClN_3O$ $[M+H]^+$: 430.2620; Determined 430.2627.

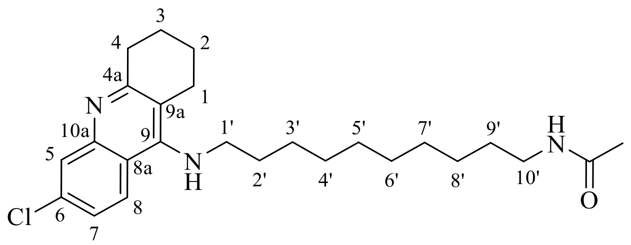

Figure S47. *N-{10-[(6-chloro-1,2,3,4-tetrahydroacridin-9-yl)amino]decyl}acetamide* (***7.m***).

N-{12-[(6-chloro-1,2,3,4-tetrahydroacridin-9-yl)amino]dodecyl}acetamide ***7.n***. Brown oil. ^{1}H NMR (300 MHz, CD_3OD): δ 1.16-1.37 (16H, m, H-2′,4′,5′,6′,7′,8′,9′,11′), 1.47 (2H, p, J = 7.2 Hz, H-10′), 1.63 (2H, p, J = 7.2 Hz, H-3′), 1.85-1.92 (4H, m, H-2,3), 1.94 (3H, s, CH_3-amide), 2.67 (2H, broad s, H-1), 2.94 (2H, broad s, H-4), 3.14 (2H, t, J = 7.1 Hz, H-12′), 3.54 (2H, t, J = 7.2 Hz, H-1′), 7.28 (1H, dd, J = 9.2 and 2.2 Hz, H-7), 7.71 (1H, d, J = 2.2 Hz, H-5), 8.06 (1H, d, J = 9.2 Hz, H-8). ^{13}C NMR (75 MHz, CD_3OD): δ 21.2 (CH_3-amide), 22.0 (C-2), 22.5 (C-3), 24.5 (C-1), 26.4 (C-2′), 26.6 (C-11′), 28.9 (C-10′), 29.0-29.3 (C4′,5′,6′,7′,8′,9′), 30.8 (C-3′), 32.4 (C-4), 39.1 (C-12′), 48.3 (C-1′), 114.9 (C-9a), 117.6 (C-8a), 123.7 (C-7), 124.5 (C-5), 125.3 (C-8), 134.5 (C-6), 146.4 (C-10a), 152.3 (C-9), 158.0 (C-4a), 171.7 (C=O). HRMS-ESI [m/z]: Calculated for $C_{29}H_{45}ClN_3O$ $[M+H]^+$: 458.2933; Determined 458.2941.

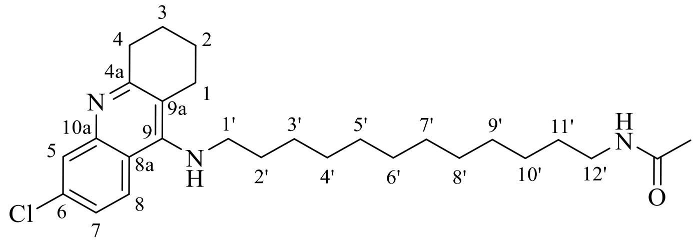

Figure S48. *N-{12-[(6-chloro-1,2,3,4-tetrahydroacridin-9-yl)amino]dodecyl}acetamide* (***7.n***).

Synthesis of *N*-formylated-9-alkylamino-1,2,3,4-tetrahydroacridine (8.a-f):

Derivative **8.a as example of NMR spectra.*

N-{8-[(1,2,3,4-tetrahydroacridin-9-yl)amino]octyl}formamide ***8.a***, Pale brown oil, yield 43 %. ^{1}H NMR (300 MHz, CD_3OD): *δ* 1.24-1.40 (8H, m, H-2′,4′,5′,7′), 1.48 (2H, p, *J* = 7.1 Hz, H-6′), 1.65 (2H, p, *J* = 7.3 Hz, H-3′), 1.93 (4H, p, *J* = 3.3 Hz, H-2,3), 2.73-2.80 (2H, m, H-4), 2.96-3.03 (2H, m, H-1), 3.19 (2H, dt, *J* = 7.1 and 0.8 Hz, H-8′), 3.55 (2H, t, *J* = 7.2 Hz, H-1′), 7.38 (1H, ddd, *J* = 8.4, 6.8 and 1.3 Hz, H-7), 7.57 (1H, ddd, *J* = 8.4, 6.8 and 1.4 Hz, H-6), 7.78 (1H, dd, *J* = 8.4 and 1.2 Hz, H-5), 8.02 (1H, s, CHO), 8.11 (1H, dd, *J* = 8.4 and 1.3 Hz, H-8). ^{13}C NMR (75 MHz, CD_3OD): *δ* 22.3 (C-3), 22.7 (C-2), 24.8 (C-4), 26.3 (C-2′), 26.4 (C-7′), 28.8 (C-6′), 28.9 (C-4′,5′), 30.8 (C-3′), 32.7 (C-1), 37.5 (C-8′), 48.3 (C-1′), 115.3 (C-9a), 119.9 (C-8a), 123.0 (C-8), 123.3 (C-7), 126.4 (C-5), 128.4 (C-6), 146.4 (C-10a), 152.0 (C-9), 157.6 (C-4a), 162.3 (CHO). HRMS-ESI [m/z]: Calculated for $C_{22}H_{32}N_3O$ $[M+H]^+$: 354.2540; Determined: 354.2582.

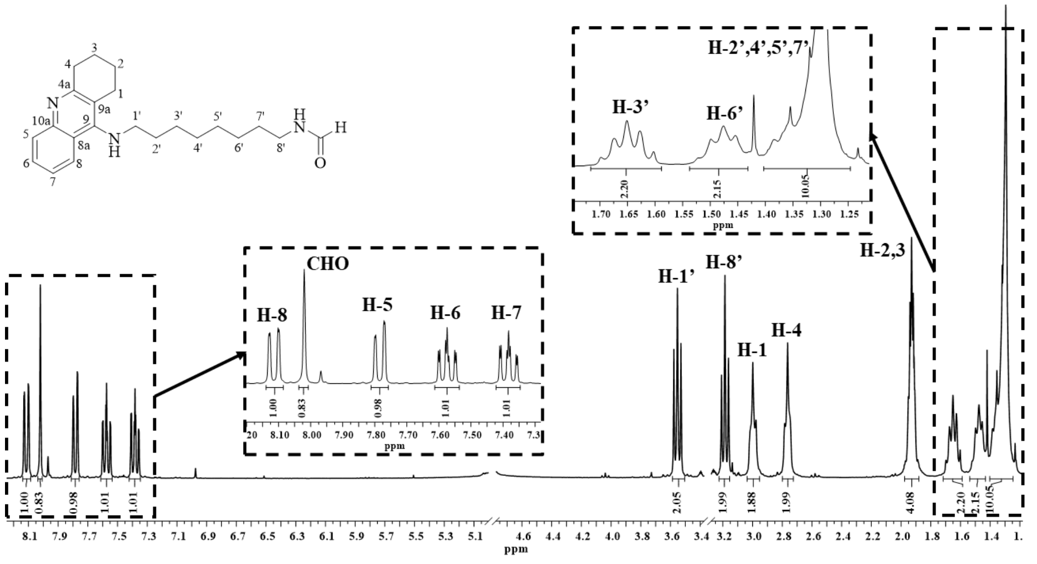

Figure S49. *^{1}H NMR spectrum of N-{8-[(1,2,3,4-tetrahydroacridin-9-yl)amino]octyl}formamide (**8.a**).*

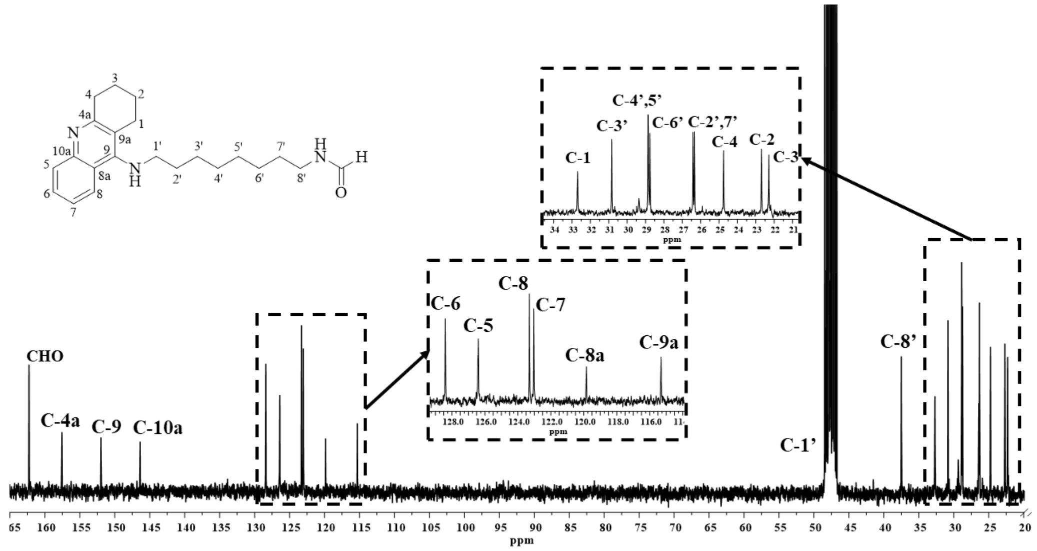

Figure S50. *^{1}H NMR spectrum of N-{8-[(1,2,3,4-tetrahydroacridin-9-yl)amino]octyl}formamide (**8.a**).*

N-{10-[(1,2,3,4-tetrahydroacridin-9-yl)amino]decyl}formamide ***8.b***. Pale brown oil, yield 88 %. ^{1}H NMR (300 MHz, CD_3OD): δ 1.23-1.40 (12H, m, H-2′,4′,5′,6′,7′,9′), 1.50 (2H, p, J = 7.3 Hz, H-8′), 1.65 (2H, p, J = 7.1 Hz, H-3′), 1.93 (4H, p, J = 3.3Hz, H-2,3), 2.73-2.80 (2H, m, H-4), 2.97-3.04 (2H, m, H-1), 3.20 (2H, t, J = 7.0 Hz, H-10′), 3.56 (2H, t, J = 7.2 Hz, H-1′), 7.38 (1H, ddd, J = 8.4, 6.8 and 1.3 Hz, H-7), 7.58 (1H, ddd, J = 8.4, 6.8 and 1.3 Hz, H-6), 7.79 (1H, dd, J = 8.5 and 1.3 Hz, H-5), 8.03 (1H, s, CHO), 8.12 (1H, dd, J = 8.5 and 1.3 Hz, H-8). ^{13}C NMR (75 MHz, CD_3OD): δ 22.3 (C-3), 22.7 (C-2), 24.8 (C-4),26.5 (C-2′), 26.5 (C-9′), 28.9 (C-8′), 28.9-29.1 (C-4′,5′,6′,7′), 30.9 (C-3′), 32.7 (C-1), 37.5 (C-10′), 48.3 (C-1′), 115.3 (C-9a), 119.8 (C-8a), 123.1 (C-8), 123.3 (C-7), 126.4 (C-5), 128.4 (C-6), 146.4 (C-10a), 152.0 (C-9), 157.5 (C-4a), 162.3 (CHO). HRMS-ESI [m/z]: Calculated for $C_{24}H_{36}N_3O$ $[M+H]^+$: 382.2853; Determined: 382.2862.

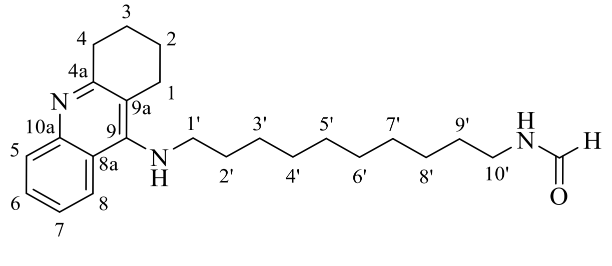

Figure S51. *N-{10-[(1,2,3,4-tetrahydroacridin-9-yl)amino]decyl}formamide (**8.b**).*

N-{12-[(1,2,3,4-tetrahydroacridin-9-yl)amino]dodecyl}formamide ***8.c***. Pale brown oil, yield 56 %. ^{1}H NMR (300 MHz, CD_3OD): δ 1.22-1.40 (16H, m, H-2′,4′,5′,6′,7′,8′,9′,11′), 1.51 (2H, p, J = 7.0 Hz, H-10′), 1.65 (2H, p, J = 7.1 Hz, H-3′), 1.93 (4H, p, J = 3.3 Hz, H-2,3), 2.72-2.81 (2H, m, H-4), 2.96-3.03 (2H, m, H-1), 3.21 (2H, t, J = 7.0 Hz, H-12′), 3.56 (2H, t, J = 7.2 Hz, H-1′), 7.39 (1H, ddd, J = 8.4, 6.8

and 1.3 Hz, H-7), 7.58 (1H, ddd, *J* = 8.4, 6.8 and 1.4 Hz, H-6), 7.79 (1H, dd, *J* = 8.5 and 1.3 Hz, H-5), 8.03 (1H, s, CHO), 8.12 (1H, dd, *J* = 8.5 and 1.3 Hz, H-8). ^{13}C NMR (75 MHz, CD_3OD): *δ* 22.3 (C-3), 22.7 (C-2), 24.7 (C-4), 26.5 (C-11′), 26.5 (C-2′), 28.9 (C-10′), 28.9-29.2 (C-4′,5′,6′,7′,8′,9′), 30.9 (C-3′), 32.6 (C-1), 37.6 (C-12′), 48.3 (C-1′), 115.2 (C-9a), 119.8 (C-8a), 123.1 (C-8), 123.3 (C-7), 126.3 (C-5), 128.5 (C-6), 146.3 (C-10a), 152.1 (C-9), 157.4 (C-4a), 162.3 (CHO). HRMS-ESI [m/z]: Calculated for $C_{26}H_{40}N_3O$ $[M+H]^+$: 410.3166; Determined: 410.3192.

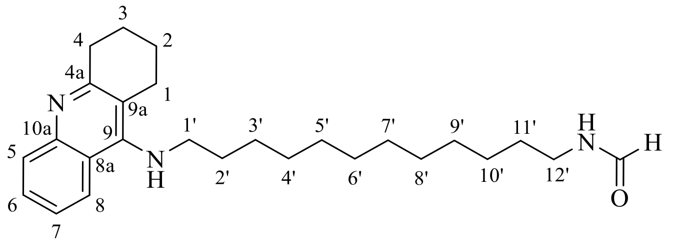

Figure S52. *N-{12-[(1,2,3,4-tetrahydroacridin-9-yl)amino]dodecyl}formamide (**8.c**).*

N-{8-[(6-chloro-1,2,3,4-tetrahydroacridin-9-yl)amino]octyl}formamide ***8.d***, Pale brown oil, yield 40 %. ^{1}H NMR (300 MHz, CD_3OD): δ 1.24-1.38 (8H, m, H-2′,4′,5′,7′), 1.47 (2H, p, *J* = 7.0 Hz, H-6′), 1.64 (2H, p, *J* = 7.4 Hz, H-3′), 1.90 (4H, p, *J* = 3.3 Hz, H-2,3), 2.70 (2H, broad s, H-1), 2.96 (2H, broad s, H-4), 3.18 (2H, t, *J* = 7.1 Hz, H-8′), 3.54 (2H, t, *J* = 7.2 Hz, H-1′), 7.30 (1H, dd, *J* = 9.2 and 2.2 Hz, H-7), 7.73 (1H, d, *J* = 2.2 Hz, H-5), 8.02 (1H, s, CHO), 8.07 (1H, d, *J* = 9.2 Hz, H-8). ^{13}C NMR (75 MHz, CD_3OD): *δ* 22.1 (C-2), 22.5 (C-3), 24.6 (C-1), 26.4 (C-2′,7′), 28.8 (C-6′), 28.9 (C-4′,5′), 30.8 (C-3′), 32.7 (C-4), 37.5 (C-8′), 48.3 (C-1′), 115.2 (C-9a), 117.9 (C-8a), 123.6 (C-7), 125.0 (C-5), 125.2 (C-8), 134.2 (C-6), 146.9 (C-10a), 152.0 (C-9), 158.6 (C-4a), 162.3 (CHO). HRMS-ESI [m/z]: Calculated for $C_{22}H_{30}ClN_3O$ $[M+H]^+$: 388.2150; Determined: 388.2162.

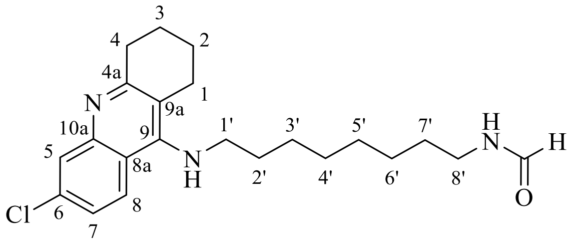

Figure S53. *N-{8-[(6-chloro-1,2,3,4-tetrahydroacridin-9-yl)amino]octyl}formamide (**8.d**).*

N-{10-[(6-chloro-1,2,3,4-tetrahydroacridin-9-yl)amino]decyl}formamide ***8.e***. Pale brown oil, yield 45 %. ^{1}H NMR (300 MHz, CD_3OD): *δ* 1.21-1.41 (12H, m, H-2',4',5',6',7',9'), 1.52 (2H, p, *J* = 7.0 Hz, H-8′), 1.65 (2H, p, *J* = 7.2 Hz, H-3′), 1.92 (4H, p, *J* = 3.3 Hz, H-2,3), 2.70-2.77 (2H, m, H-4), 2.95-3.01 (2H, m, H-1), 3.18-3.25 (2H, t, *J* = 6.9 Hz, H-10′), 3.57 (2H, t, *J* = 7.1 Hz, H-1′), 7.33 (1H, dd, *J* = 9.1 and 2.2 Hz, H-7), 7.75 (1H, d, *J* = 2.2 Hz, H-5), 8.03 (1H, s, CHO), 8.11 (1H, d, *J* = 9.1 Hz, H-8). ^{13}C NMR (75 MHz, CD_3OD): *δ* 22.2 (C-3), 22.6 (C-2), 24.6 (C-4), 26.4 (C-2′), 26.5 (C-9′), 28.9 (C-8′), 28.9-29.2

(C-4′,5′,6′,7′), 30.8 (C-3′), 32.8 (C-1), 37.6 (C-10′), 48.3 (C-1′), 115.3 (C-9a), 118.0 (C-8a), 123.6 (C-7), 125.1 (C-8), 125.2 (C-5), 134.2 (C-6), 147.1 (C-10a), 152.1 (C-9), 158.8 (C-4a), 162.3 (CHO). HRMS-ESI [m/z]: Calculated for $C_{24}H_{34}ClN_3O$ $[M+H]^+$: 416.2463; Determined: 416.2491.

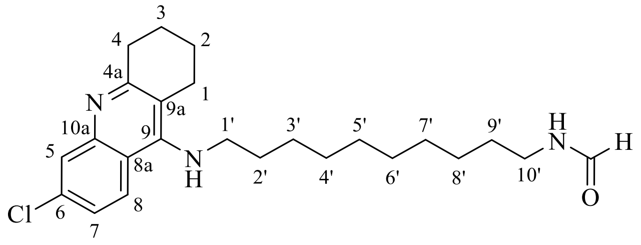

Figure S54. *N-{10-[(6-chloro-1,2,3,4-tetrahydroacridin-9-yl)amino]decyl}formamide (**8.e**).*

N-{12-[(6-chloro-1,2,3,4-tetrahydroacridin-9-yl)amino]dodecyl}formamide ***8.f***. Pale brown oil, yield 57 %. ^{1}H NMR (300 MHz, CD_3OD): δ 1.22-1.40 (16H, m, H-2',4',5',6',7',8',9',11'), 1.50 (2H, p, *J* = 7.0 Hz, H-10′), 1.65 (2H, p, *J* = 7.3 Hz, H-3′), 1.93 (4H, p, *J* = 3.3 Hz, H-2,3), 2.69-2.77 (2H, m, H-4), 2.94-3.02 (2H, m, H-1), 3.21 (2H, t, *J* = 7.0 Hz, H-12′), 3.58 (2H, t, *J* = 7.1 Hz, H-1′), 7.33 (1H, dd, *J* = 9.1 and 2.2 Hz, H-7), 7.75 (1H, d, *J* = 2.2 Hz, H-5), 8.03 (1H, s, CHO), 8.11 (1H, d, *J* = 9.1 Hz, H-8). ^{13}C NMR (75 MHz, CD_3OD): δ 22.2 (C-3), 22.6 (C-2), 24.6 (C-4), 26.4 (C-11′), 26.5 (C-2′), 28.9 (C-10′), 28.9-29.1 (C-4′,5′,6′,7′,8′,9′), 30.8 (C-3′), 32.8 (C-1), 37.5 (C-12′), 48.3 (C-1′), 115.3 (C-9a), 118.0 (C-8a), 123.6 (C-7), 125.1 (C-5), 125.2 (C-8), 134.2 (C-6), 147.1 (C-10a), 152.1 (C-9), 158.8 (C-4a), 162.3 (CHO). HRMS-ESI [m/z]: Calculated for $C_{26}H_{38}ClN_3O$ $[M+H]^+$: 444.2776; Determined: 444.2804.

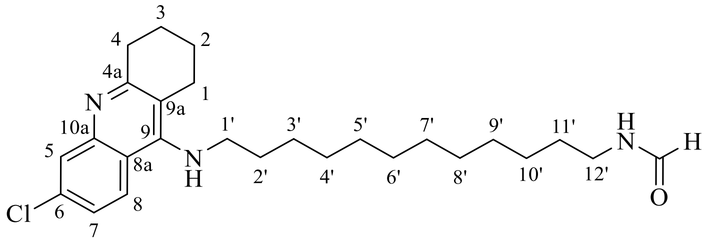

Figure S55. *N-{12-[(6-chloro-1,2,3,4-tetrahydroacridin-9-yl)amino]dodecyl}formamide (**8.f**).*

Synthesis of compounds 9.a-d:

Derivative* ***9.b* *as example of NMR spectra.*

2,5-bis({3-(1,2,3,4-tetrahydroacridin-9-yl)amino]propyl}amino)cyclohexa-2,5-diene-1,4-dione ***9.a***. Pale brown oil, yield 34 %. ^{1}H NMR (500 MHz, (CD$_3$OD): δ 1.93-1.97 (8H, m, H-2,3), 2.16 (4H, t, J = 6.6 Hz, H-2′), 2.69 (4H, broad s, H-4), 2.99 (4H, broad s, H-1), 3.35-3.38 (4H, m, H-3′), 4.05 (4H, t, J = 6.6 Hz, H-1′), 5.04 (2H, s, H-3′′), 7.54 (2H, ddd, J = 8.5, 6.9 and 1.3 Hz, H-7), 7.73 (2H, dd, J = 8.5 and 1.3 Hz, H-5), 7.79 (2H, ddd, J = 8.5, 6.8 and 1.1 Hz, H-6), 8.32 (2H, d, J = 8.5 Hz, H-8) ^{13}C NMR (125 MHz, (CD$_3$OD): δ 21.3 (C-2,3), 23.7 (C-4), 28.1 (C-1), 28.4 (C-2′), 39.3 (C-3′), 45.3 (C-1′), 91.8 (C-3′′), 111.9 (C-9a), 115.7 (C-8a), 119.0 (C-5), 124.9 (C-7,8), 132.5 (C-6), 138.6 (C-10a), 150.7 (C-4a), 151.6 (C-2′′), 156.5 (C-9), 177.5 (C-1′′). HRMS-ESI [m/z]: Calculated for $C_{38}H_{43}N_6O_2$ $[M+H]^+$: 615.3442; Determined: 615.3428.

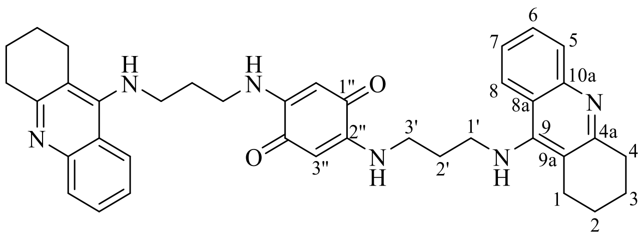

Figure S56. *2,5-bis({3-[(1,2,3,4-tetrahydroacridin-9-yl)amino]propyl}amino)cyclohexa-2,5-diene-1,4-dione (**9.a**).*

2,5-dichloro-3,6-bis({3-[(1,2,3,4-tetrahydroacridin-9-yl)amino]propyl}amino)cyclohexa-2,5-diene-1,4-dione ***9.b***. Light brown solid, m.p. 145-150 °C, yield 44 %. ^{1}H NMR (500 MHz, CD$_3$OD): δ 1.93-1.97 (8H, m, H-2,3), 2.18 (4H, p, J = 6.5 Hz, H-2′), 2.70 (4H, broad s, H-4), 2.98 (4H, broad s, H-1), 3.95 (4H, t, J = 6.6 Hz, H-3′), 4.05 (4H, t, J = 6.4 Hz, H-1′), 4.64 (4H, broad s, NH), 7.51 (4H, t, J = 7.7 Hz, H-7), 7.70 (4H, d, J = 7.7 Hz, H-5), 7.76 (4H, t, J = 7.7 Hz, H-6), 8.30 (4H, d, J = 8.6 Hz, H-8). ^{13}C NMR (125 MHz, CD$_3$OD): δ 20.6 (C-3), 21.7 (C-2), 23.8 (C-4), 28.5 (C-1), 31.1 (C-2′), 41.2 (C-3′), 44.8 (C-1′), 112.2 (C-9a), 116.0 (C-8a), 119.6 (C-5), 124.8 (C-7,8), 132.2 (C-6), 134.7 (C-3′′), 139.3 (C-10a), 145.1 (C-2′′), 151.3 (C-4a), 156.0 (C-9), 177.3 (C-1′′). HRMS-ESI [m/z]: Calculated for $C_{38}H_{41}Cl_2N_6O_2$ $[M+H]^+$: 683.2663; Determined 683.2692.

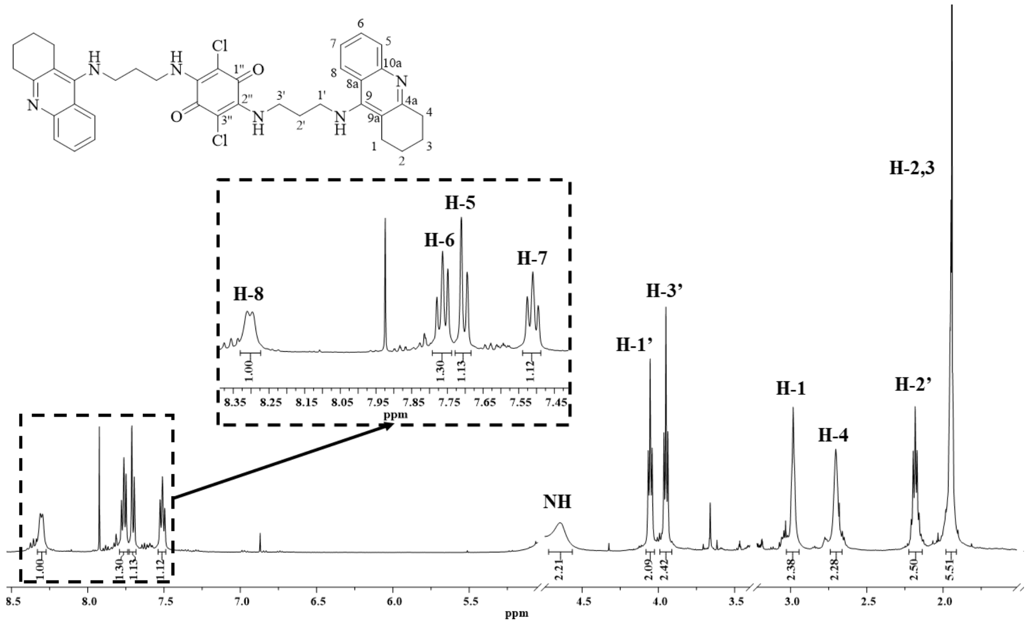

Figure S57. *^{1}H NMR spectrum of 2,5-dichloro-3,6-bis({3-[(1,2,3,4-tetrahydroacridin-9-yl)amino]propyl}amino)cyclohexa-2,5-diene-1,4-dione (**9.b**).*

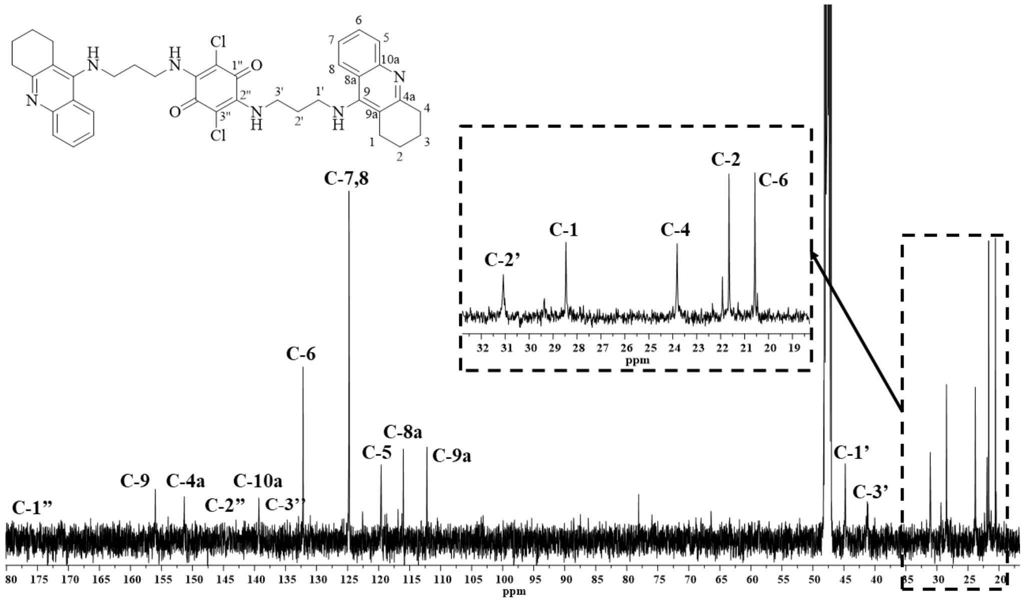

Figure S58. *^{13}C NMR spectrum of 2,5-dichloro-3,6-bis({3-[(1,2,3,4-tetrahydroacridin-9-yl)amino]propyl}amino)cyclohexa-2,5-diene-1,4-dione (**9.b**).*

2,5-bis({3-[(6-chloro-1,2,3,4-tetrahydroacridin-9-yl)amino]propyl}amino)cyclohexa-2,5-diene-1,4-dione ***9.c***. Pink solid, m.p. 220-225 °C, yield 19 %. ^{1}H NMR (300.13 MHz, DMSO-d$_6$): δ 1.76-1.84 (8H, m, H-2,3), 1.88 (4H, p, J = 6.8 Hz, H-2′), 2.64 (4H, broad s, H-4), 2.90 (4H, broad s, H-1), 3.20 (4H, dd, J = 12.9 and 6.5 Hz, H-3′), 3.59-3.67 (4H, m, H-1′), 5.19 (2H, s, H-3′′), 7.39 (2H, d, J = 8.8 Hz, H-7), 7.73 (2H, d, J = 2.2 Hz, H-5), 7.85 (2H, t, J = 6.5 Hz, NH-benzoquinone), 8.20 (2H, d, J = 9.2 Hz, H-8). ^{13}C NMR (75.47 MHz, DMSO-d$_6$): δ 22.0 (C-2,3), 28.9 (C-2′), 31.2 (C-4), 31.8 (C-1), 40.0 (C-3′),

45.5 (C-1′), 92.6 (C-3′′), 114.6 (C-9a), 119.3 (C-8a), 124.5 (C-7), 124.7 (C-5), 126.6 (C-8), 134.6 (C-6), 148.1 (C-10a), 151.6 (C-9), 156.4 (C-4a), 170.4 (C-2′′), 177.7 (C-1′′). HRMS-ESI [m/z]: Calculated for $C_{38}H_{41}Cl_2N_6O_2$ [M+H]+: 683.2663; Determined 683.2651.

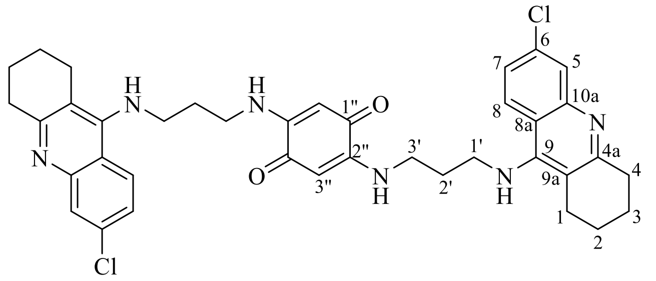

Figure S59. *2,5-bis({3-[(6-chloro-1,2,3,4-tetrahydroacridin-9-yl)amino]propyl}amino)cyclohexa-2,5-diene-1,4-dione (**9.c**).*

2,5-dichloro-3,6-bis({3-[(6-chloro-1,2,3,4-tetrahydroacridin-9-yl)amino]propyl}amino)cyclohexa-2,5-diene-1,4-dione **9.d** Brown solid, m.p. 165-175 ˚C (deg), yield 29 %. ^{1}H NMR (300 MHz, DMSO-d_6): δ 1.73-1.81 (8H, m, H-2,3), 1.87 (4H, p, J = 6.5 Hz, H-2′), 2.61-2.68 (4H, broad s, H-4), 2.81-2.89 (4H, broad s, H-1), 3.50 (4H, t, J = 6.5 Hz, H-3′), 3.67-3.77 (4H, m, H-1′), 5.91 (2H, broad s, NH-α), 7.31 (2H, dd, J = 9.1 and 2.3 Hz, H-7), 7.67 (2H, d, J = 2.3 Hz, H-5), 8.13 (2H, d, J = 9.1 Hz, H-8). ^{13}C NMR (75 MHz, DMSO-d_6): δ 22.5 (C-3), 22.9 (C-2), 25.6 (C-4), 32.3 (C-2′), 33.6 (C-1), 41.8 (C-1′), 44.8 (C-3′), 115.9 (C-9a), 118.6 (C8a), 124.0 (C-7), 125.8 (C-8), 126.5 (C-5), 133.2 (C-6), 135.4 (C-3′′), 147.4 (C-10a), 150.9 (C-9), 159.1 (C-4a), 170.5 (C-2′′), 171.7 (C-1′′). HRMS-ESI [m/z]: Calculated for $C_{38}H_{39}Cl_4N_6O_2$ [M+H]$^+$: 751.1883; Determined 751.1864.

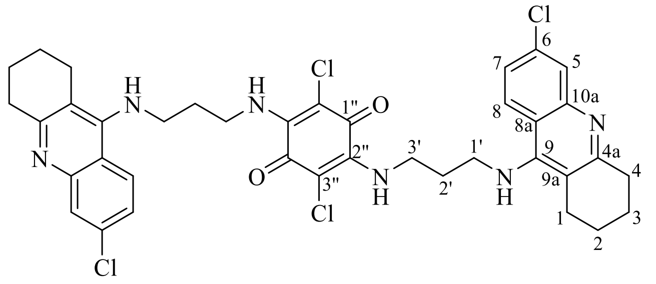

Figure S60. *2,5-dichloro-3,6-bis({3-[(6-chloro-1,2,3,4-tetrahydroacridin-9-yl)amino]propyl}amino)cyclohexa-2,5-diene-1,4-dione (**9.d**).*

Synthesis of N^1,N^{12}-bis(7-chloroquinolin-4-yl)dodecane-1,12-diamine (12):

N^1,N^{12}-bis(7-chloroquinolin-4-yl)dodecane-1,12-diamine **12**, Beige solid, 190-194 ˚C (188-190 ˚C)[17], yield 53 %. ^{1}H NMR (500 MHz, $CDCl_3$): *δ* 1.27-1.33 (8H,m, H-5′,6′,7′,8′), 1.33-1.40 (4H, m, H-4′; 9′), 1.46 (4H, p, *J* = 7.3 and 6.8 Hz, H-2′,11′), 1.76 (4H, p, *J* = 7.3 Hz, H-3′,10′), 3.31 (4H, td, *J* = 7.2 and 5.1 Hz, H-1′,12′), 4.98 (2H, broad s, NH), 6.42 (2H, d, *J* = 5.4 Hz, H-3), 7.37 (2H, dd, *J* = 8.9 and 2.2 Hz, H-6), 7.66 (2H, d, *J* = 8.9 Hz, H-5), 7.96 (2H, d, *J* = 2.2 Hz, H-8), 8.54 (2H, d, *J* = 5.4 Hz, H-2). ^{13}C NMR (125 MHz, $CDCl_3$): δ 27.1 (C-2′,11′), 28.9 (C-3′,10′), 29.3 (C-4′,9′), 29.5 (C-6′,7′), 29.5 (C-5′,8′), 43.3 (C-1′,12′), 99.1 (C-3), 117.1 (C-4a), 120.8 (C-5), 125.3 (C-6), 128.8 (C-8), 134.9 (C-8a), 149.0 (C-7), 149.8 (C-4), 152.0 (C-2). HRMS-ESI [m/z]: Calculated for $C_{30}H_{37}Cl_2N_4$[M+H]$^+$: 523.2390; Determined: 523.2410.

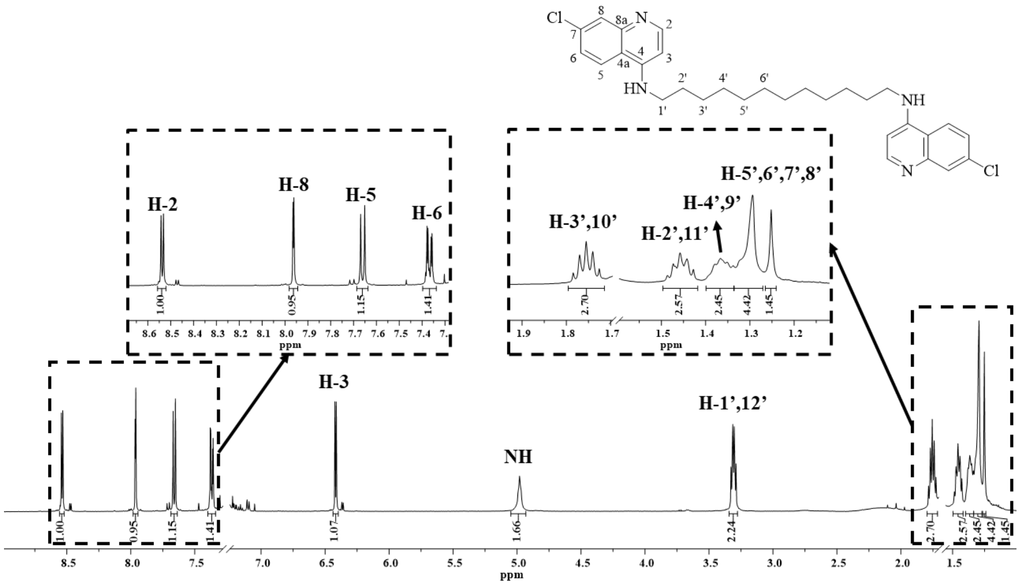

Figure S61. *^{1}H NMR spectrum of N^1,N^{12}-bis(7-chloroquinolin-4-yl)dodecane-1,12-diamine (**12**).*

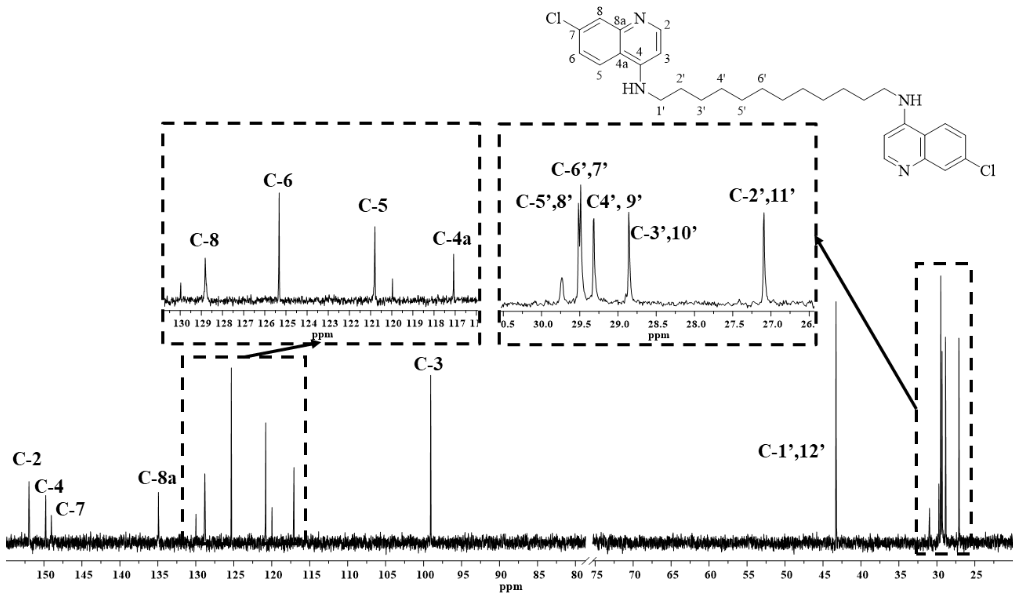

Figure S62. *^{13}C NMR spectrum of N[1],N[12]-bis(7-chloroquinolin-4-yl)dodecane-1,12-diamine (**12**).*
